# Identification of a two-component regulatory system involved in antimicrobial peptide resistance in *Streptococcus pneumoniae*

**DOI:** 10.1371/journal.ppat.1010458

**Published:** 2022-04-08

**Authors:** Aissatou Maty Diagne, Anaïs Pelletier, Claire Durmort, Agathe Faure, Kerstin Kanonenberg, Céline Freton, Adeline Page, Frédéric Delolme, Jaroslav Vorac, Sylvain Vallet, Laure Bellard, Corinne Vivès, Franck Fieschi, Thierry Vernet, Patricia Rousselle, Sébastien Guiral, Christophe Grangeasse, Jean-Michel Jault, Cédric Orelle

**Affiliations:** 1 Molecular Microbiology and Structural Biochemistry (MMSB), UMR 5086 CNRS/University of Lyon, Lyon, France; 2 Institute of Structural Biology (IBS), UMR 5075 CNRS/University of Grenoble-Alpes, Grenoble, France; 3 Protein Science Facility, SFR BioSciences, CNRS, UMS3444, INSERM US8, University of Lyon, Lyon, France; 4 Laboratoire de Biologie Tissulaire et Ingénierie Thérapeutique (LBTI), UMR 5305 CNRS/University of Lyon, Lyon, France; Lunds universitet Medicinska fakulteten, SWEDEN

## Abstract

Two-component regulatory systems (TCS) are among the most widespread mechanisms that bacteria use to sense and respond to environmental changes. In the human pathogen *Streptococcus pneumoniae*, a total of 13 TCS have been identified and many of them have been linked to pathogenicity. Notably, TCS01 strongly contributes to pneumococcal virulence in several infection models. However, it remains one of the least studied TCS in pneumococci and its functional role is still unclear. In this study, we demonstrate that TCS01 cooperates with a BceAB-type ABC transporter to sense and induce resistance to structurally-unrelated antimicrobial peptides of bacterial origin that all target undecaprenyl-pyrophosphate or lipid II, which are essential precursors of cell wall biosynthesis. Even though *tcs01* and *bceAB* genes do not locate in the same gene cluster, disruption of either of them equally sensitized the bacterium to the same set of antimicrobial peptides. We show that the key function of TCS01 is to upregulate the expression of the transporter, while the latter appears the main actor in resistance. Electrophoretic mobility shift assays further demonstrated that the response regulator of TCS01 binds to the promoter region of the *bceAB* genes, implying a direct control of these genes. The BceAB transporter was overexpressed and purified from *E*. *coli*. After reconstitution in liposomes, it displayed substantial ATPase and GTPase activities that were stimulated by antimicrobial peptides to which it confers resistance to, revealing new functional features of a BceAB-type transporter. Altogether, this inducible defense mechanism likely contributes to the survival of the opportunistic microorganism in the human host, in which competition among commensal microorganisms is a key determinant for effective host colonization and invasive path.

## Author summary

*Streptococcus pneumoniae* is a commensal bacterium of the human nasopharynx that can switch to an invasive pathogen causing a variety of diseases, leading to over one million deaths worldwide each year. The sophisticated strategies that allow *S*. *pneumoniae* to survive in various environments within the human body are still poorly understood. One of the most widespread tools that enable bacteria to sense environmental changes and to promote adaptative responses by modulating gene expression are two-component regulatory systems (TCS). TCS01 was identified as an important virulence factor, and understanding its biological function is key to comprehend bacterial pathogenesis. In this study, we demonstrated that this TCS upregulates the expression of an ABC transporter that mediates resistance to bacterial antimicrobial peptides targeting cell wall synthesis. Because competition among microorganisms is a key element for host colonization and persistence, our findings contribute to explain the potent role of TCS01 in bacterial survival within the human host.

## Introduction

*Streptococcus pneumoniae*, also known as the pneumococcus, is a common commensal colonizer of the human nasopharynx [1,2]. Under certain circumstances, however, it can invade distant sites and become pathogenic, causing infections such as sinusitis and otitis media, but also life-threatening invasive diseases such as community-acquired pneumonia, meningitis and septicemia. As a consequence, the pneumococcus causes each year over one million deaths worldwide, mostly in children under 5 years old, elderly and immunocompromised individuals [3,4]. As for most pathogenic bacteria, antibiotic resistance is an increasing issue in pneumococcal infections [5]. In 2017, the WHO listed *S*. *pneumoniae* as one of the 12 priority pathogens for research and development of new antibiotics.

The ability to switch from a harmless commensal to a pathogen has required the microorganism to evolve sophisticated strategies to survive in various environments within the human body [2,6]. Pneumococcal survival and pathogenicity are governed by dynamic interactions with other commensal bacterial species and resident host cells [7], as well as the ability to evade the host inflammatory and immune responses [2]. During its path from the upper respiratory tract to deeper tissues, the survival of the pneumococcus relies on its ability to sense environmental changes and to adapt accordingly its gene expression patterns, notably those related to virulence, metabolism and transport [2]. Pneumococcal gene expression is tightly controlled by a range of sensorial tools [1], among which two-component regulatory systems (TCS) are the most widespread and conserved in bacteria [8–10].

These transduction systems typically consist of a transmembrane Histidine Kinase (HK) that senses a signal via an extracellular domain and subsequently phosphorylates a cognate cytoplasmic Response Regulator (RR), the two HK and RR encoding genes being often organized in operons [11,12]. Classically, an HK dimer autophosphorylates on its C-terminal cytoplasmic domain in response to a specific stimulus. Then, the HK transfers the phosphoryl group to a conserved aspartate residue of its cognate RR. This induces a conformational change in the RR that modulates its interaction with a partner (DNA, RNA or protein) and consequently the biological response of the bacterial cell. In total, 13 TCS and one orphan RR have been identified in *S*. *pneumoniae* [13]. Several studies analyzing the impact of individual inactivation of each TCS system on *S*. *pneumoniae* pathogenicity have shown that ten out of these 13 TCS contribute to pneumococcal virulence [14]. Amongst them, TCS05 (CiaRH) and TCS12 (ComDE) are the best studied, while TCS01 is the least characterized [9]. Strikingly, the HK belonging to TCS01 lacks an extracellular sensory domain, thus questioning the nature of the signal triggering HK autophosphorylation and its functional role. Pioneering work has nevertheless evidenced that disruption of *tcs01* genes results in a dramatic attenuation of the growth (by 10^5^ fold) in a mouse respiratory tract infection model [13]. In addition, several independent studies using various other models confirmed the implication of this system in virulence [15,16]. Very recently, Reinoso-Vizcaino and colleagues reported that TCS01 is critical for *S*. *pneumoniae* survival in influenza-infected cells and proposed that it plays a role in resistance to acidic and oxidative stress associated with viral co-infection [17]. Based on these findings the authors renamed TCS01 as SirRH (for stress-induced response), SirH being the HK and SirR the RR. However, how TCS01 contributes to the pneumococcal physiopathology in absence of viral infection remains unknown.

In this study, we demonstrate that TCS01 functions in tight cooperation with a BceAB-type ABC transporter to provide resistance to a range of antimicrobial peptides targeting cell wall biosynthesis.

## Results

### Function of TCS01: homology-based hypothesis

A similarity search was performed based on the primary sequences of the HK and RR present in TCS01 of *Streptococcus pneumoniae*. We first applied a Blast protein search to *Bacillus subtilis*, which is the best-characterized member of Gram-positive bacteria. This analysis revealed that the three closest HK homologues in *B*. *subtilis* are YxdK, BceS and YvcQ (S1 Table). By using the same approach for the RR of TCS01, we found that its closest homologues are the RRs that are encoded from the same operons of the aforementioned genes, i.e. YxdJ, BceR and YvcP, respectively (S1 Table). The three identified TCS proteins in *B*. *subtilis* belong to specialized systems involved in antimicrobial peptide resistance, where each TCS functions in cooperation with an ABC transporter belonging to the BceAB subfamily (originally named for Bacitracin efflux), to both sense and promote resistance to antimicrobial peptides [18–21]. In *B*. *subtilis* and most of the Firmicutes, these TCS genes are adjacent to their cognate ABC transporter genes [22]. In contrast, there is no ABC genes in the immediate vicinity of the *tcs01* operon in *S*. *pneumoniae*. Hence, we searched for putative pneumococcal homologues of the three *B*. *subtilis* ABC transporters. BceAB-type transporters are composed of two subunits that are encoded in operons, i.e. the Nucleotide-Binding Domains (NBD) and the transmembrane domains (TMD). Because the sequences of the NBDs are highly conserved in the ABC superfamily, and possibly not discriminative enough, we used the sequence of the TMD to perform the pBLAST search. For each of the three *B*. *subtilis* TMDs of the ABC transporters, the only similar protein that emerged in *S*. *pneumoniae* was Spr0813/SPD_0805/SP_0913 (nomenclatures in R6/D39/TIGR4 strains, respectively; S2 Table). As its counterparts from *B subtilis*, this ABC transporter was previously shown to be involved in antimicrobial peptide resistance [23,24]. Consistent with the identification of Spr0813 as a member of the BceAB subfamily in *S*. *pneumoniae*, its predicted topology is similar to that of the three TMD proteins from *B*. *subtilis*; such a topology is unique among the ABC superfamily with a large extracellular domain (~200 residues) between the predicted transmembrane helices 7 and 8, presumably folding as an independent domain (S1 Fig). Overall, the high amino acid sequence similarities between these systems (S3 Table) suggest that TCS01 might function with the Spr0812/Spr0813 ABC transporter to provide antimicrobial peptide resistance in *S*. *pneumoniae*, albeit the operon encoding TCS01 is located 0.64 Mb away from the operon carrying these ABC transporter genes [23] (S2 Fig). Consistent with this hypothesis, Dintner and colleagues performed phylogenetic and co-evolution analyses of ~250 *Firmicutes* BceAB-type ABC transporters and TCS components, and predicted a functional link for a number of them that are not in the same genome neighborhood, including our pair of interest in *S*. *pneumoniae* [25]. Strikingly, whereas many Firmicutes bacteria contain up to six of these BceAB-type transporters with both distinct and overlapping substrate specificities [25], *S*. *pneumoniae* contains a single one.

### Function of *spr1473*/*spr1474* (*tcs01*) and *spr0812*/*spr0813* (*bceAB*) genes in antimicrobial peptide resistance

**Fig 1:**
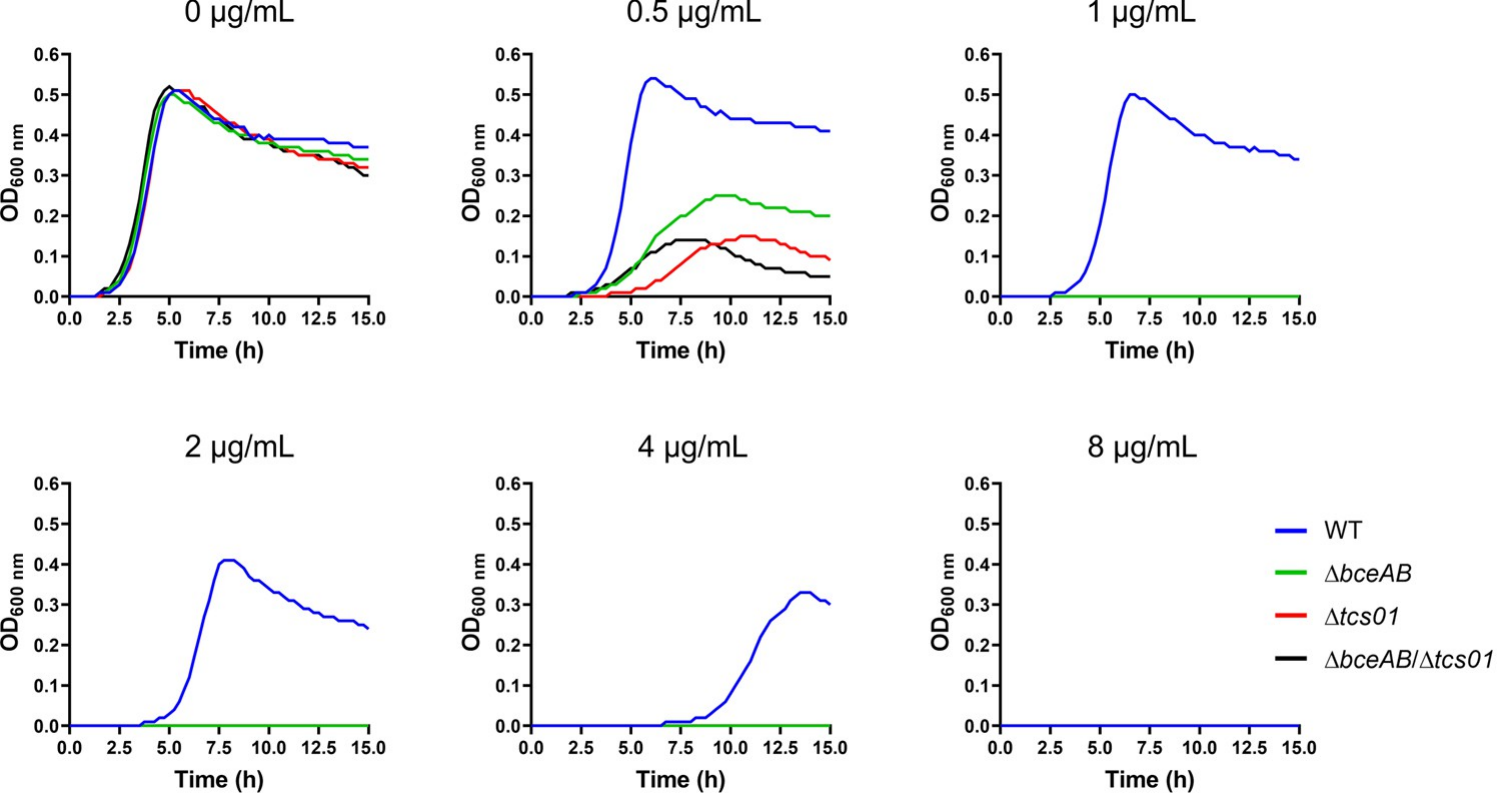
Growth of wild-type and mutant strains in the absence or presence of various concentrations of bacitracin. The bacitracin concentrations are indicated on top of the graphs. Cultures were performed in microplates. This representative experiment of 4 biological replicates shows the average of technical duplicates grown in a 96-well microplate.

We generated deletion mutant strains of the genes of interest encoding the ABC transporter and/or the TCS in the R6 strain of *S*. *pneumoniae* (hereafter named Δ*bceAB*, Δ*tcs01*, or Δ*bceAB*/*tcs01* for simplicity) (Table 1). Because the R6 strain lacks a polysaccharide capsule and is avirulent, its safety and genetic malleability makes it convenient for investigation of pneumococcal biology [26]. Importantly, the deletion of *bceAB* and/or *tcs01* genes did not impair the bacterial growth in Todd-Hewitt broth (Fig 1, top left panel). However, the growth of the mutant strains was severely impaired in the presence of bacitracin (Fig 1). To analyze more precisely the implication of *tcs01* and *bceAB* genes in antimicrobial peptide (AMP) resistance, we used the broth microdilution method to determine the Minimum Inhibitory Concentrations (MICs) of a large range of AMPs for the wild-type and mutant strains. The inactivation of the ABC transporter genes notably sensitized the bacterium to a number of antimicrobial peptides, including bacitracin, nisin, actagardin and planosporicin, for which the MICs were reduced by 8 to 16-fold in comparison to the wild-type strain (Table 2). Previous work solely identified two antimicrobial peptides to which it confers resistance (bacitracin and nisin), while instead a truncated mutant of this transporter (lacking 2 of the ten transmembrane helices) conferred resistance to vancoresmycin [23,24]. Our results are consistent with the observation that the Δ*BceAB* mutant in the pathogenic D39 strain exhibits an increased susceptibility to bacitracin and nisin [24] and additionally show that BceAB also confers resistance to a large range of AMPs of various bacterial origins: actagardin and NAI-802 (*Actinoplanes sp*.), planosporicin (*Planomonospora sp*.), microbisporicin (*Microbispora sp*.) and NAI-857 (*Streptomyces sp*.). Remarkably, the inactivation of the TCS genes had exactly the same effects as the inactivation of the ABC transporter genes, i.e. Δ*bceAB* and Δ*tcs01* cells displayed the same sensitivity levels to the aforementioned AMPs (Table 2). Furthermore, the double knock-out mutant strain (Δ*bceAB*/*tcs01*) was equally sensitive to the strains bearing a single operon inactivation (Table 2). Similar effects were observed in the pathogenic D39 strain, since the integrity of both the *bceAB* and *tcs01* genes were necessary for AMP resistance (Table 2). Taken together, these observations strongly suggest that TCS01 and BceAB function in cooperation to provide resistance to a range of antimicrobial peptides with unrelated structures (S3 Fig). Interestingly, all these compounds identified here target precursors of the cell wall, i.e. undecaprenyl-pyrophosphate (UPP or C55-PP) for bacitracin or lipid II for the others [27–33].

**Table 1:** *S*. *pneumoniae* strains used in this study.

Strains	Relevant properties	Source or ref.
R6	Wild type	[26]
D39	Wild type	[34]
R800	R6 *rpsL1; Str*^*R*^	[35]
Δ*bceAB*	R6 Δ*spr0812 spr0813*::*Cat*^*R*^ D39 Δ*SPD_0804 SPD_0805*::*Cat*^*R*^	This work
Δ*tcs01*	*R6* Δ*spr1473 spr1474*::*Kan*^*R*^ *D39* Δ*SPD_1445 SPD_1446*::*Kan*^*R*^	This work
Δ*bceAB*/Δ*tcs01*	R6 Δ*spr0812 spr0813*::*Cat*^*R*^ */* Δ*spr1473 spr1474*::*Kan*^*R*^ D39 Δ*SPD_0804 SPD_0805*::*Cat*^*R*^ */* Δ*SPD_1445 SPD_1446*::*Kan*^*R*^	This work
Δ*bceA*::*kan-rpsL*	R800, Δ*spr0812*::*Kan-rpsL*	This work
*bceA-kan-rpsL* ^a^	R800, *spr0812 Kan-rpsL*	This work
*bceA-gfp*	R800, *spr0812-gfp*	This work
Δ*hk01*::*kan-rpsL*^a^	R800, Δ*spr1473*::*Kan-rpsL*	This work
Δ*hk01*	R800, Δ*spr1473*	This work
Δ*hk01 bceA-kan-rpsL*^a^	R800, Δ*spr1473*, *spr0812 Kan-rpsL*	This work
Δ*hk01 bceA-gfp*	R800, Δ*spr1473*, *spr0812-gfp*	This work
P_*comX*_*-kan-rpsL*^a^	R800 *rpsL*::*rpsL1*, Δ*spr0565Nter*::*P1*::*PcomR-comR*, *cpsN-cpsO*::*PcomX-kan-rpsL*	[36]
P_*comX*_*-rr01-hk01*^a^	R800 *rpsL*::*rpsL1*, Δ*spr0565Nter*::*P1*::*PcomR-comR*, *cpsN-cpsO*::*PcomX-rr01-hk01*	This work
P_*comX*_*-rr01-hk01* Δ*hk01*::*kan-rpsL*^a^	R800 *rpsL*::*rpsL1*, Δ*spr0565Nter*::*P1*::*PcomR-comR*, *cpsN-cpsO*::*PcomX-rr01-hk01*, Δ*spr1473*::*kan-rpsL*	This work
P_*comX*_*-rr01-hk01* Δ*hk01*	R800 *rpsL*::*rpsL1*, Δ*spr0565Nter*::*P1*::*PcomR-comR*, *cpsN-cpsO*::*PcomX-rr01-hk01*, Δ*spr1473*	This work
P_*comX*_*-rr01-hk01* Δ*hk01* Δ*bceAB*::*kan-rpsL*^a^	R800 *rpsL*::*rpsL1*, Δ*spr0565Nter*::*P1*::*PcomR-comR*, *cpsN-cpsO*::*PcomX-rr01-hk01*, Δ*spr1473*, Δ*spr0812-spr0813*::*kan-rpsL*	This work
P_*comX*_*-rr01-hk01* Δ*hk01* Δ*bceAB*	R800 *rpsL*::*rpsL1*, Δ*spr0565Nter*::*P1*::*PcomR-comR*, *cpsN-cpsO*::*PcomX-rr01-hk01*, Δ*spr1473*, Δ*spr0812-spr0813*	This work

^a^ These strains are intermediate constructs that were not directly used in our experiments

**Table 2:** Minimum Inhibitory Concentrations (μg/ml) of various antimicrobial peptides (AMPs) against the R6 and D39 strains.

**R6 strain**					
**AMPs**	WT	Δ*bceAB*	Δ*tcs01*	Δ*bceAB/tcs01*	Fold sensitivity displayed by the KO strains
actagardin	32	2	2	2	16
bacitracin	8	1	1	1	8
nisin	3.2	0.4	0.4	0.4	8
Planosporicin (NAI-97)	16	2	2	2	8
NAI-802	16	2	2	2	8
Microbisporicin (NAI-107)	0.032	0.008	0.008	0.008	4
NAI-857	4	2	2	2	2
gramicidin	0.016	0.016	0.016	0.016	1
ramoplanin	0.02	0.02	0.02	0.02	1
vancomycin	0.5	0.5	0.5	0.5	1
mastoporan	32	32	32	32	1
colistin	256	256	256	256	1
**D39 strain**					
**AMPs**	WT	Δ*bceAB*	Δ*tcs01*	Δ*bceAB/tcs01*	Fold sensitivity diplayed by the KO strains
bacitracin	4	1	1	1	4
nisin	3.2	0.2	0.2	0.2	16
actagardin	32	2	2	2	16
vancomycin	0.25	0.25	0.25	0.25	1
ramoplanin	0.03	0.03	0.03	0.03	1

Experiments were performed with two to four biological replicates, each with technical duplicates.

### TCS01 is involved in the upregulation of the *bceAB* genes

**Fig 2:**
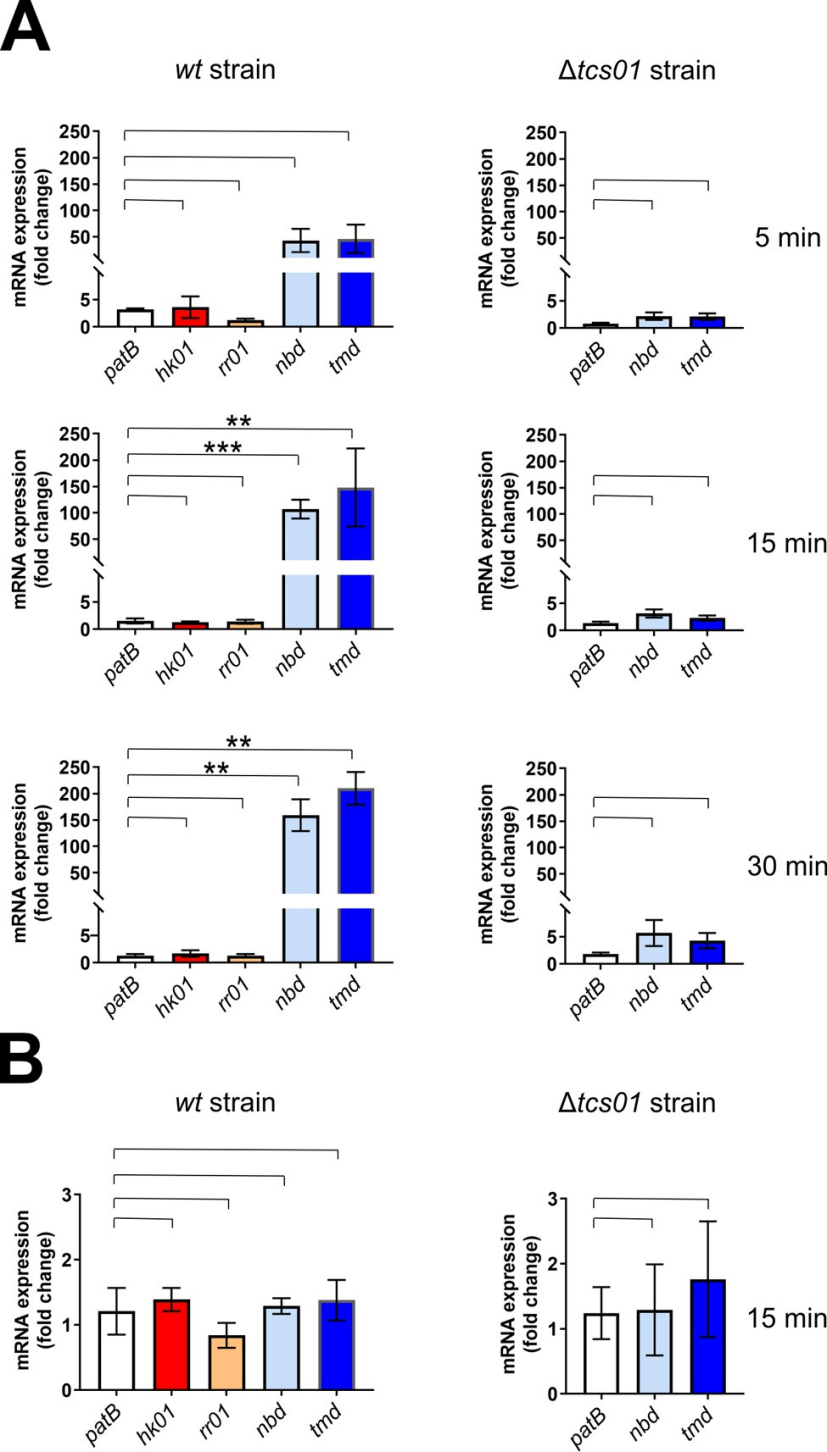
Quantification of gene expression in *S*. *pneumoniae* in the presence of bacitracin (A) or vancomycin (B). (A) After bacitracin treatment (1 μg/ml for 5, 15 and 30 min), the amount of mRNAs extracted from D39 strains was quantified by qPCR relatively to a control without bacitracin. The ABC genes are in blue tone colors, while the tcs*01* genes are shown in red and orange colors. The *patB* gene was used as a control that does not respond to bacitracin treatment in the experiment. Data are the average of biological replicates (n = 6 for time 5 and 30 min; n = 8 for time 15 min) and the standard deviation of the mean is shown. Statistical significance was calculated by Student´s t-test with Welch’s correction between the conditions indicated with brackets. In the absence of label above the bracket, the *p* value is above 0.05 and considered not significant, whereas statistically significant differences are indicated with ** (*p*≤0.01) and *** (*p*≤0.001). (B) After vancomycin (375 ng/ml for 15 min) treatment, the amount of mRNAs extracted from D39 strains was quantified by qPCR relatively to a control without vancomycin. Data are the average of biological replicates (n = 8) and the standard deviation of the mean is shown. Statistical significance was calculated by Student´s t-test with Welch’s correction as in A.

Because the inactivation of either *tcs01 or bceAB genes* abrogated the resistance to antimicrobial peptides, we aimed at clarifying the contribution of each partner in the sensing and resistance mechanism. In most BceAB-type systems, the ABC transporter is not only responsible for AMP resistance but is also required for signaling the presence of AMPs to the cognate TCS [22]. This is explained by the fact that initial AMP recognition likely occurs via its binding to the extracellular domain of the BceAB-type transporter (S1 Fig), since a domain swap experiment between two such transporters from *Staphylococcus aureus* exchanged their specificities [37]. Nevertheless, in some bacteria such as *S*. *aureus*, a dedicated sensing ABC transporter is present to promote the induction of a second transporter that in return mediates the actual resistance [37]. We first sought to determine by qPCR whether the *tcs01* and *bceAB* genes are upregulated in the presence of antimicrobial peptides. The *patB* gene, encoding a subunit of a multidrug ABC transporter [38], was used as a negative control that does not respond to bacitracin treatment in the experiment. In the presence of bacitracin, a strong upregulation of the *bceAB* genes was observed in the wild-type strain while the transcription of the *tcs01* genes was unaffected (Fig 2A). In the Δ*tcs01* knockout mutant strain, bacitracin treatment failed to upregulate the transcription of the *bceAB* genes. These observations strongly suggest that TCS01 is involved in the upregulation of the *bceAB* genes in response to the presence of bacitracin. As another negative control, wild-type cells were treated with vancomycin, for which no resistance is provided by the resistance module (Table 2), and no upregulation of the *bceAB* genes was observed (Fig 2B). Altogether, these results confirm the mechanistic coupling of BceAB and TCS01 and furthermore suggest that the AMP signaling mechanism is a prerequisite for establishing resistance by the ABC transporter.

**Fig 3:**
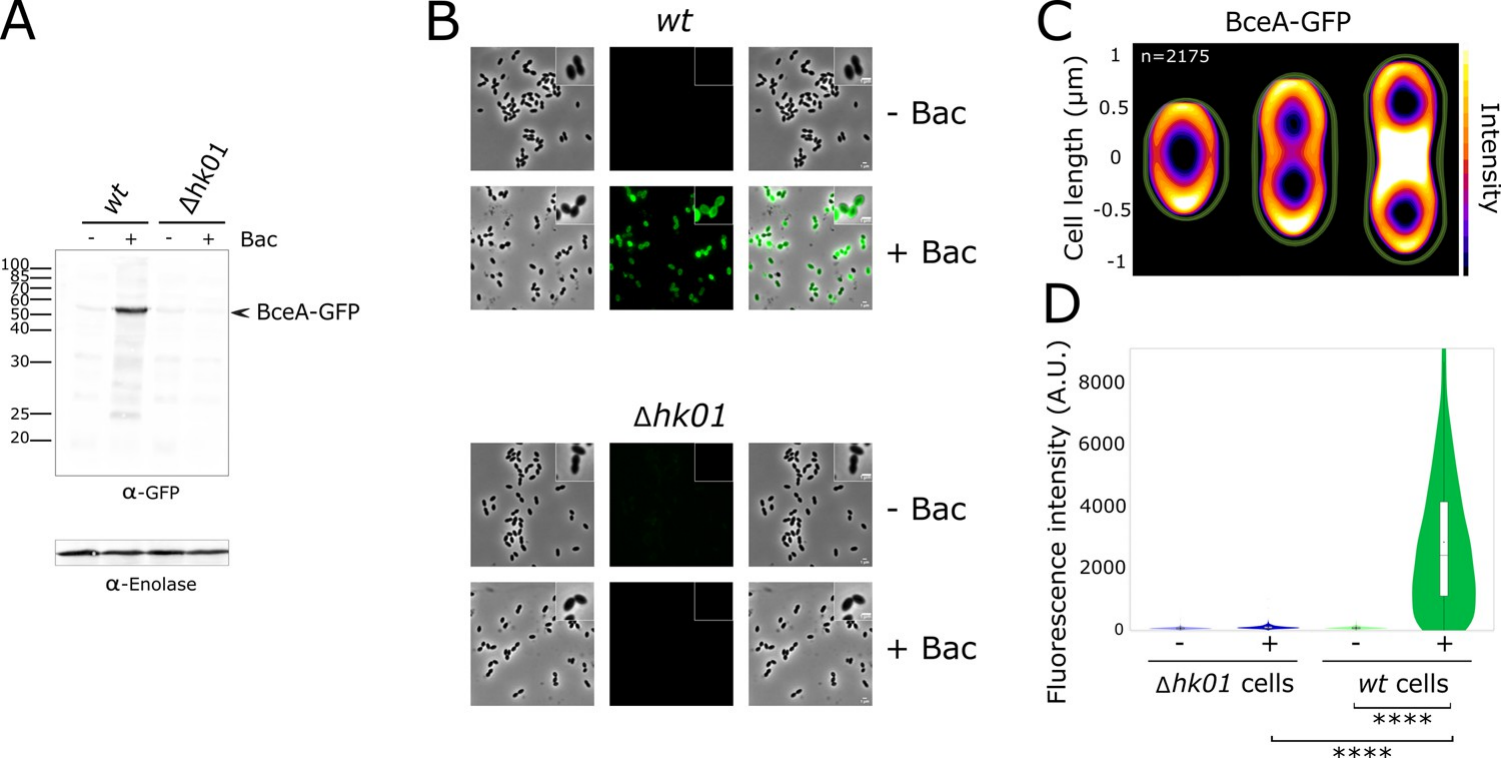
Analysis of BceAB-GFP overexpression in *S*. *pneumoniae* upon bacitracin treatment. These experiments were conducted in R800 strains in which the *hk01* gene was either intact (wt) or deleted (Δ*hk01*). In both strains, the *gfp* gene was fused to the gene encoding the nucleotide-binding domain of BceAB. (A) Western blot analysis of BceAB-GFP expression in wt or Δ*hk01* cells previously bacitracin-treated (+ Bac) or untreated (- Bac). Crude bacterial extracts are revealed with anti-GFP and anti-Enolase antibodies, the later providing a protein loading control. The arrow indicates the position of GFP fused to the nucleotide-binding domain of BceAB. (B) Fluorescence microscopy of *S*. *pneumoniae* cells analyzing the bacitracin-dependent expression of BceAB-GFP in wt or Δ*hk01* strains. Phase contrast (left panel), GFP fluorescent signal (middle panel) and overlays between phase contrast and GFP images (right panel) are shown. Enlargement is shown on upper right corners of each panel. Scale bar, 1 μm. (C) Heat maps representing the localization patterns of BceAB-GFP during the cell cycle. The n-values represent the number of cells analyzed in a single representative experiment. The images and n values are representative of experiments performed in triplicate. (D) Violin plots showing the distribution of cellular fluorescence intensities in individual cells showed in panel B. The boxes in the violin plots indicate the 25^th^ to the 75^th^ percentile and the whiskers indicate the minimum and maximum value. The mean and the median are indicated with a dot and a line in the box, respectively. The P value (****, p<0.0001) was derived from a Mann-Whitney test. A total of 10, 442 cells were analyzed.

Next, we investigated whether the strong upregulation of *bceAB* transcription resulted in higher protein levels. We performed this analysis by fusing the *gfp* gene to the 3’-end of the gene encoding the nucleotide-binding domain BceA at its endogenous chromosomal location. This construct was engineered in the R800 strain, a derivative of the R6 strain that contain a streptomycin resistant mutation to facilitate the selection of the constructs of interest [35]. Importantly, the GFP fusion did not impair the functionality of BceAB since the engineered strain displayed comparable resistance for bacitracin, nisin and actagardin as compared to the wild-type strain (Table 3). Cells were challenged with bacitracin, and the subsequent upregulation of BceA was clearly visible by Western Blot using an Anti-GFP antibody (Fig 3A). To complement this global analysis, we further quantified the response of individual cells by fluorescence microscopy. Bacitracin treatment strongly activated the expression of BceA-GFP, as indicated by a large increase in fluorescence for a majority of cells (Fig 3B). To exclude that the fluorescence originates from NBDs that dissociated from the transmembrane domains of the transporter, we analysed the subcellular localization of GFP and its fluorescence was mostly at the vicinity of the membrane (Fig 3C), as confirmed with additional staining of the membrane with FM4-64 (S4 Fig). Finally, the upregulation of BceA-GFP in the presence of bacitracin was clearly dependent on the integrity of the *hk01* gene, again confirming the functional coupling of TCS01 and the BceAB-type transporter (Fig 3A, 3B and 3D).

**Table 3:** Minimum Inhibitory Concentrations of several antimicrobial peptides against the R800 strains.

MIC (μg/ml)	WT	*bce*AB-*gfp*	Δ*bceAB*	Δ*hk01*	Δ*hk01*-P_*comX*_-*hk01*	Δ*bceAB*-Δ*hk01*-P_*comX*_-*hk01*
Bacitracin	4	4	1	1	256	1
Nisin	1.6	1.6	0.05	0.05	1.6	0.05
Actagardin	32	32	4	4	32	4
ramoplanin	0.02	0.02	0.02	0.02	0.02	0.02
Vancomycin	0.25	0.25	0.25	0.25	0.25	0.25

At least biological triplicates (each with technical duplicates) were performed for each condition.

### TCS01 directly controls the expression of the *bceAB* genes

**Fig 4:**
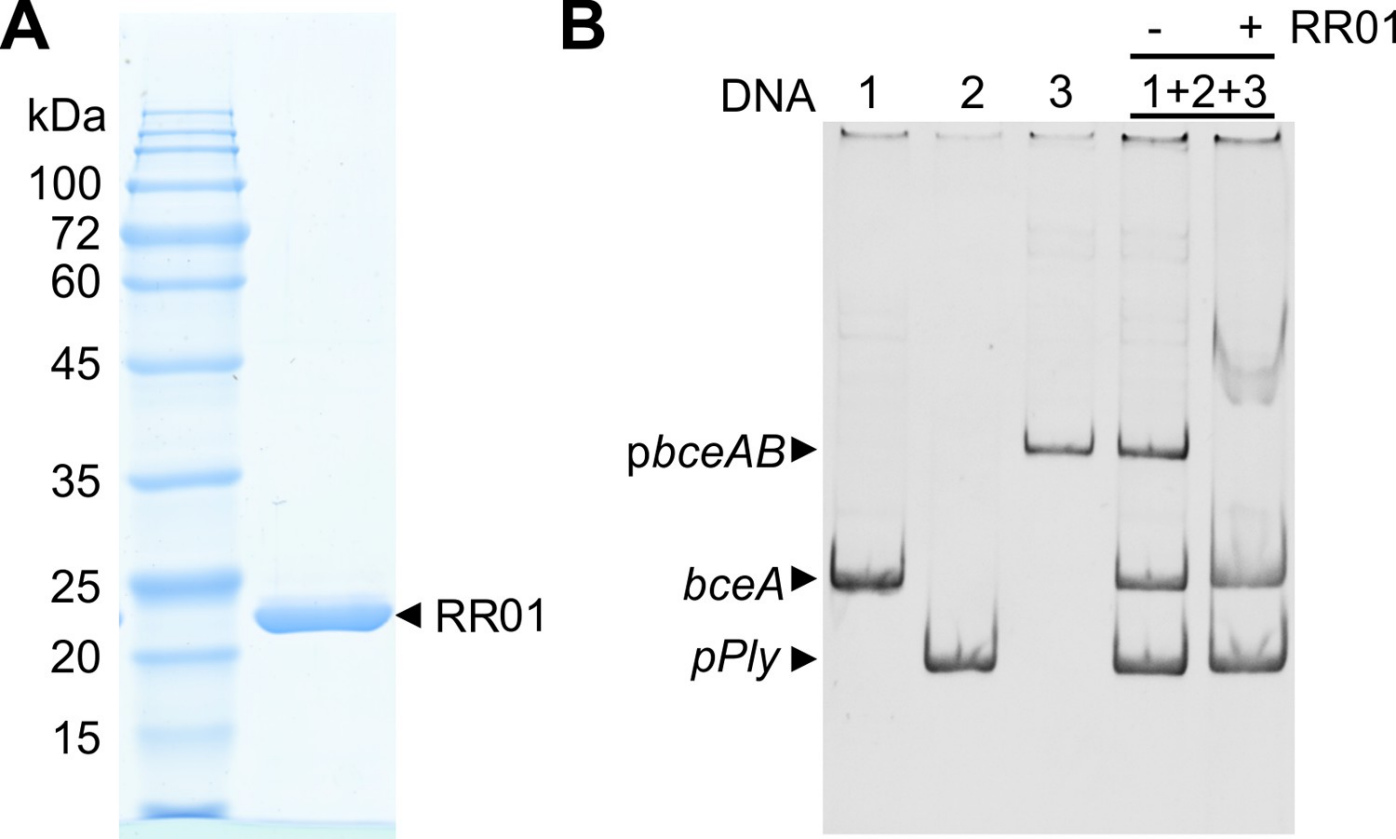
Electrophoretic mobility shift assays showing the binding of RR01 to the promoter of the *BceAB* genes. (A) SDS-PAGE of the purified RR01 (D52E) mutant. (B) electrophoretic mobility shift assays. In this experiment, 100 ng of DNA fragments covering the promoter region of BceAB genes (p*bceAB*, 557 pb), the promoter region of the pneumolysin *ply gene* (p*Ply*, 250-bp) and an internal region of the bceA gene (*bceA*, 379-bp) were incubated with an excess of the purified RR01 (D52E) mutant. A representative experiment of three independent ones is shown here.

As major mediators of signal transduction in bacteria, two-component systems can be involved in complex gene regulatory networks through branched pathways, cross-talks, and cross-regulation via connector proteins [39]. We sought to address whether TCS01 directly or indirectly controls the expression of the *bceAB* genes. The consensus binding sequence for the response regulators associated with *bceAB*-type genes has been identified as TNACA-N4-TGTAA with an AT-rich central 4-nucleotide spacer [25]. This signature is actually present 227 bases before the start codon of the *bceA* gene. To experimentally prove that RR01 is indeed able to bind this promoter region, we overexpressed and purified from *E*. *coli* a mutant of this protein containing a phosphomimetic of the conserved aspartate (D52E) [40]. Although unphosphorylated RR proteins can also bind their target promoter, phosphorylation was observed to increase their affinity [41]. We then tested by Electrophoretic Mobility Shift Assays (EMSA) its interaction with a DNA fragment encompassing the promoter region. Two other DNA fragments were used as negative controls, belonging to the *bceA* gene or the promoter region of the pneumolysin *ply* gene. Because RR01 was only able to bind the promoter region of the *bceAB* genes (Fig 4), we could thus conclude that TCS01 directly controls the expression of the *bceAB* genes.

### TCS01-dependent protein expression in *S*. *pneumoniae*

**Fig 5:**
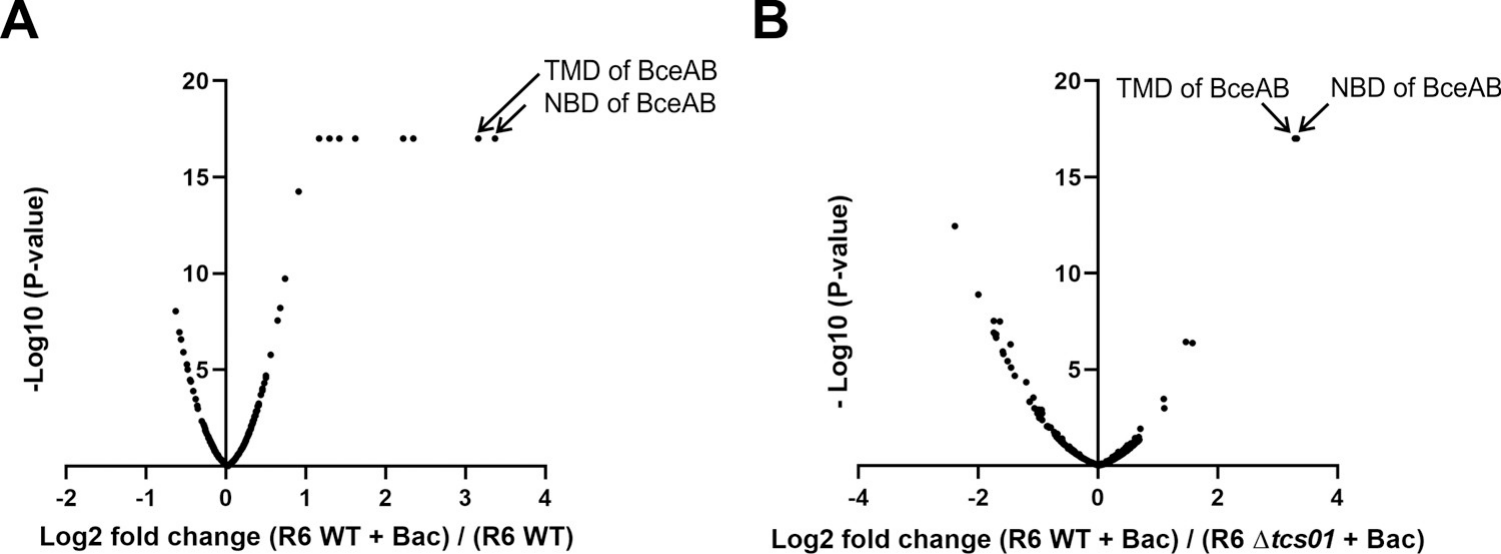
Proteomic analysis of wild-type and Δ*tcs01* R6 strains with or without bacitracin treatment. Data correspond to one biological replicate (experiment 2, see Material and Methods), while data from another biological replicate (experiment 1, see Material and Methods) are shown in S5 Fig. (A) volcano plot showing proteins differentially expressed in the wild-type strain upon bacitracin (Bac) treatment (1 μg/ml for 45 min). (B) volcano plot showing proteins differentially expressed in wild-type strain as compared to the Δ*tcs01* in the presence of bacitracin. Proteins are significantly overrepresented when Log2 (fold change) > 1 and–Log10 (P-value) > 1.3. Proteins are significantly underrepresented when Log2 (fold change) < -1 and–Log10 (P-value) > 1.3.

We next sought to determine whether TCS01 upregulates the expression of other proteins possibly involved in antimicrobial peptide resistance. As mentioned above, a dedicated sensing transporter in *Staphylococcus aureus* is for instance present to promote the overexpression of a second transporter that actually mediates the resistance [37]. To address this question, we employed a quantitative proteomics approach based on isobaric Tandem Mass Tag (TMT) labeling [42] to analyze the relative amount of proteins in the wild-type and Δ*tcs01* strains before and after bacitracin treatment. The global nature of the proteome analysis allows the unbiased identification and quantification of proteins that are differentially expressed under the control of TCS01 and upon cell exposure to bacitracin. In order to get the best proteome coverage as possible, the TMT-labeled proteins were processed according to different strategies. The experiment 1 was performed with fractionation of the labeled proteins prior to LC-MS/MS analysis. The experiment 2 was done with no pre-fractionation but the labeled proteins were separated with a longer C18 column for LC-MS/MS analysis (see Material and Methods for details). With these methodologies, we were able to detect respectively 1146 and 1024 proteins, covering about 50–55% of the *S*. *pneumoniae* proteome [26]. Bacitracin treatment of the wild-type strain resulted in a 7 ± 3 fold accumulation of BceAB (data for experiment 2 are shown in Fig 5A; data for experiment 1 are displayed in S5 Fig). In contrast, the same treatment had no effect on BceAB protein levels in the Δ*tcs01* strain (0.99 ± 0.09 fold on average), confirming the previously described qPCR and cell fluorescence results. The only proteins significantly and consistently overexpressed upon bacitracin treatment in the wild-type strain but not in the Δ*tcs01* strain were the NBD and the TMD of BceAB (Fig 5B). These observations showed that TCS01 is essential for relaying the presence of antimicrobial peptides and the subsequent overexpression of the *BceAB* genes. To summarize, our proteomics analysis showed that first, no other ABC transporter is involved in this TCS01-dependent bacitracin resistance pathway and second, the sole overexpression of BceAB is presumably sufficient to confer substantial AMP resistance to *S*. *pneumoniae*.

### Complementation of the *hk01* gene restored or increased antimicrobial peptide resistance

To rule out unexpected polarity effects and to rigorously confirm that the sensitivity phenotypes of the Δ*hk01* mutant are indeed caused by disruption of this gene, we sought to complement the R800 Δ*hk01* mutant strain with an ectopic expression of the *rr01*-*hk01* genes, which are overlapping in the genome. This strain (R800-Δ*hk01*-P_*comX*_-*rr01-hk01*) showed equal or surprisingly even higher levels of resistance as compared to the WT strain (64-fold more resistance to bacitracin) (Table 3). To better understand the determinants of this resistance, we analyzed by quantitative proteomics the overexpressed proteins when treating the cells at 32 μg/mL of bacitracin (concentration at which the wild-type strain could not survive) as compared to 1 μg/mL. We observed that the abundance of 81 proteins were increased by at least 2-fold, but the two most overexpressed proteins were the TMD (BceB) and the NBD (BceA) of the ABC transporter (S6 Fig and S1 Data, sheet 11). Nonetheless, some of the other overexpressed proteins (e.g. an unknown membrane protein SPD_0995, phosphate transporters, SecG, alkaline shock protein, murein transglycosilase, pneumococcal surface proteins etc.) may also directly or indirectly contribute to the resistance. To confirm that the BceAB-type transporter is a major player in bacitracin resistance, we deleted the *bceAB* genes in this super-resistant strain (R800-Δ*bceAB*-Δ*hk01*-P_*comX*_-rr01-*hk01*) and we observed a complete loss of resistance (Table 3).

### Functional properties of the BceAB transporter from *S*. *pneumoniae*

**Fig 6:**
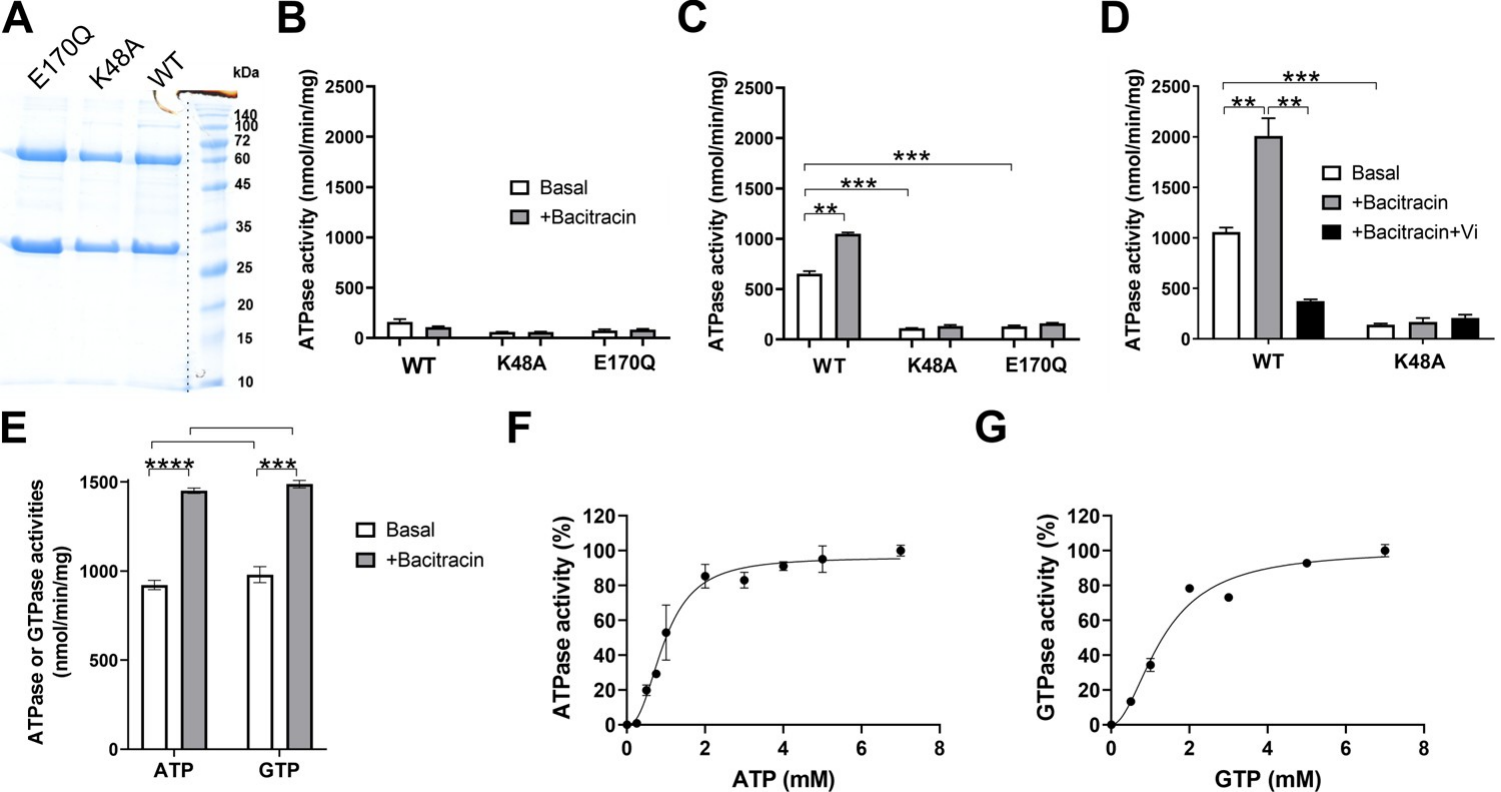
Functional features of BceAB after purification and reconstitution in liposomes. (A) SDS-PAGE of the purified wild-type BceAB and catalytic mutants (K48A and E170Q). (B) ATPase activities of the transporters in LMNG detergent. In this experiment and in panels C-E, 5 mM of nucleotides and 300 μg/mL of bacitracin were used, where indicated. (C) ATPase activities of the transporters after reconstitution in liposomes. These activities were calculated according to the total amount of proteins added to the reconstitution mixture. (D) ATPase activities of the transporters after reconstitution in liposomes and further separation of the proteoliposomes by sucrose gradient. Where indicated, othovanadate (Vi) was used at 100 μM. (E) comparison of the ATPase and GTPase activities of the wild-type protein. (F) and (G), ATPase and GTPase activities of the wild-type transporter as a function of ATP and GTP concentrations, respectively. Data were fitted with positive cooperativity ((F), n_H_ = 2.4, K_M_ = 1 mM and (G), n_H_ = 1.9, K_M_ = 1.3 mM). Data shown are one representative experiment of at least two independent experiments and error bars indicate the standard deviation of triplicates. Statistical significance in panels C-E was calculated by Student´s t-test with Welch’s correction between the conditions indicated with brackets. Statistically significant differences are indicated with ** (*p*≤0.01) and *** (*p*≤0.001).

Although deletion of *bceAB* genes caused a strong sensitivity to a number of antimicrobial peptides, we sought to provide biochemical evidence for the interaction of these peptides with the transporter. Furthermore, little is known about the functional properties of BceAB-type transporters, since no member of this subfamily has been successfully studied *in vitro* in an active form. Although these transporters were early assumed to be efflux pumps, they were recently proposed to rather function as mechanotransducers, i.e. that they do not move the substrate across the membrane but instead uses intracellular ATP hydrolysis to perform mechanical work in the periplasm [43,44]. *In vivo*, mutations that prevent ATP hydrolysis, thus abolishing antimicrobial peptide protection, also abolish the overexpression of the ABC transporter [20,45]. Therefore, a fully functional ABC transporter seems to be required to propagate the signal transduction via the TCS. Yet, the ATPase features of BceAB transporters and how antimicrobial peptides modulate their activity are still unknown. As a first step towards BceAB purification, an efficient overexpression was devised. Although the C41(DE3) and C43(DE3) strains are often more favorable for the expression of membrane proteins [46], only the BL21(DE3) strain significantly expressed the BceAB transporter from *S*. *pneumoniae* [47]. A quality control of membrane protein production in a functional state is the ability of mild detergents to solubilize them [48,49], and LMNG successfully extracted the transporter from *E*. *coli* membranes [47]. After purification of the transporter by Ni-NTA affinity chromatography, we measured the ATPase activity of the wild-type transporter. As controls, we also purified and measured the ATPase activity of transporters in which the conserved lysine in the Walker A motif or the catalytic glutamate adjacent to the Walker B motif were mutated. These mutations are known to abolish or strongly abrogate the ATPase activity of ABC transporters, as shown for the multidrug transporter BmrA [50,51]. The comparison of these activities strongly suggest that BceAB purified in LMNG detergent is poorly active and that the measured ATPase activities originate from contaminants of the preparations (Fig 6A and 6B). This is in line with the fact that the BceAB transporter from *Bacillus subtilis* was found inactive when purified in N-dodecyl-β-D-maltoside [52].

**Fig 7:**
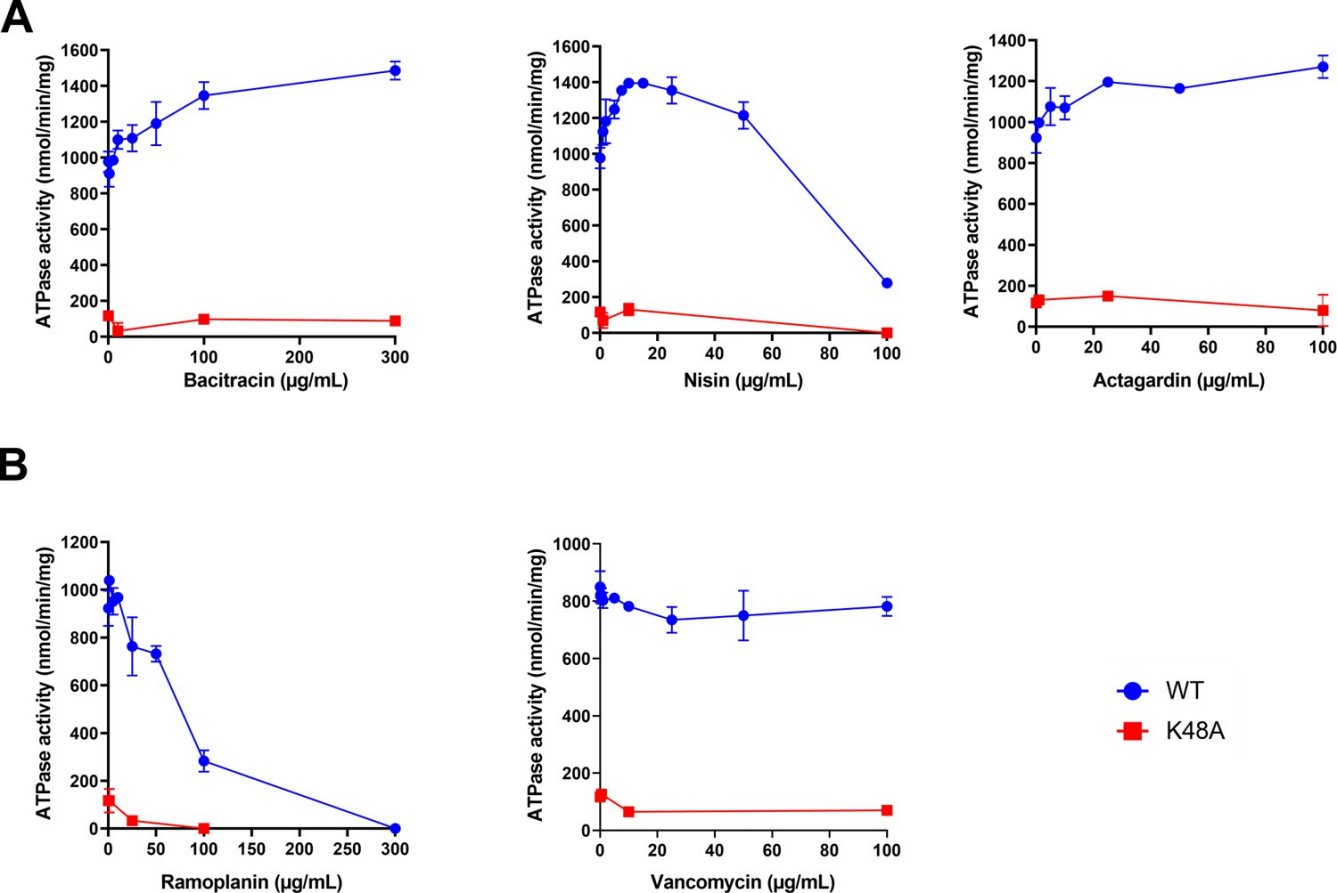
ATPase activity of BceAB in the presence of various antimicrobial peptides. (A) antimicrobial peptides to which BceAB confer resistance. (B) antimicrobial peptides to which BceAB does not confer resistance. Data shown are one representative experiment of at least two independent experiments and error bars indicate the standard deviation of triplicates.

However, after reconstitution in liposomes, the wild-type BceAB from *S*. *pneumoniae* recovered a substantial ATPase activity that is stimulated by bacitracin, reaching an activity in the range of 0.6–1 μmol of ATP hydrolyzed/min/mg, whereas both mutants showed a low and poorly-stimulated activity (Fig 6C). A further separation of the proteoliposomes on sucrose gradient allowed to increase the specific activity of the wild-type transporter up to 2 μmol ATP hydrolyzed/min/mg in the presence of bacitracin (Fig 6D), most likely by removing aggregates or transporters that fail to reconstitute properly. The antimicrobial peptides to which the transporter confer resistance to were able to stimulate the WT ATPase activity between 1.3 and 2-fold (Figs 6D and 7A). In contrast, vancomycin did not significantly affect its ATPase activity, whereas ramoplanin had a clear inhibitory effect (Fig 7B). This indicates that ramoplanin does bind to the transporter but acts as an inhibitor, which may explain -at least partially- why the transporter does not confer resistance to it despite that this compound targets lipid II.

Next, because the multidrug ABC transporter PatA/PatB from *S*. *pneumoniae* was shown to preferentially use GTP as energy source [38], we investigated the nucleotide specificity of the BceAB transporter. The levels of ATPase and GTPase activities displayed by the transporter are relatively similar (Fig 6E), although the ATPase displayed a more pronounced positive cooperativity (Fig 6F and 6G). This suggests that the BceAB can be energized equally well by ATP and GTP *in vivo*, since the concentrations of these nucleotides are in the same mM range in *S*. *pneumoniae* [38].

## Discussion

**Fig 8:**
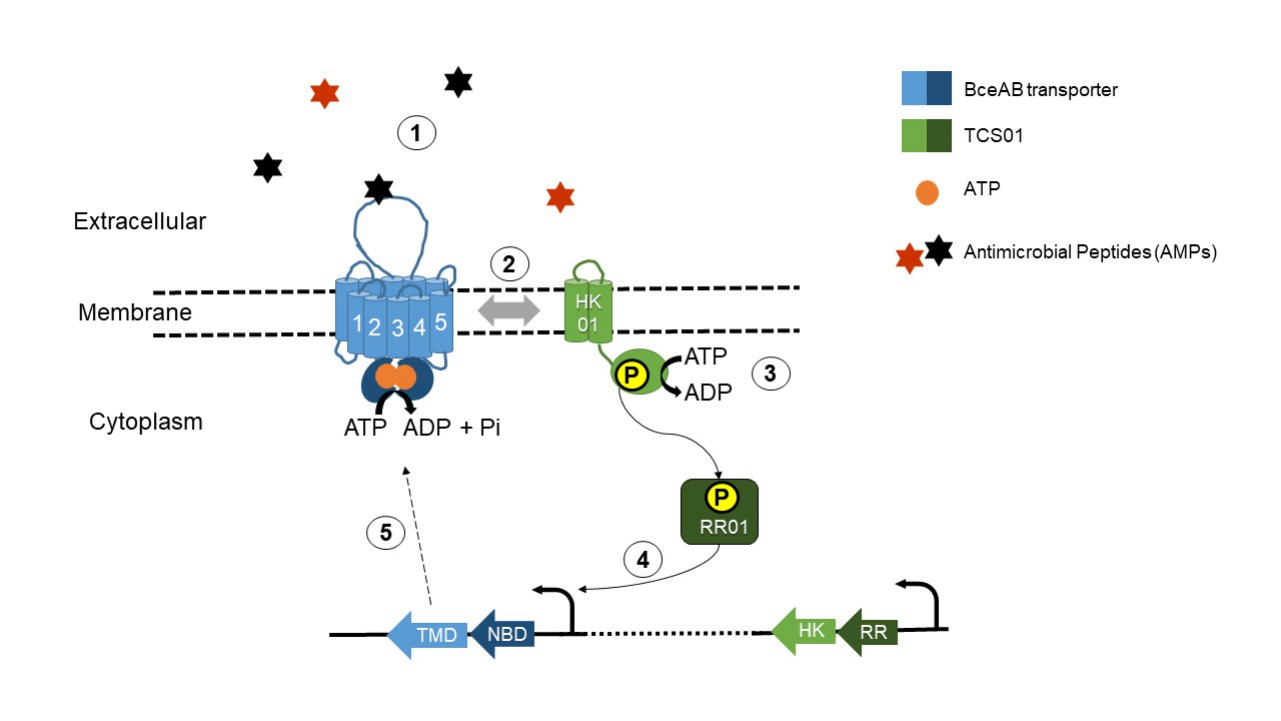
Functional module involving TCS01 in *Streptococcus pneumoniae*. The BceAB/TCS01 module is able to sense the presence of antimicrobial peptides targeting undecaprenylpyrophosphate (UPP) or lipid II (step 1). The AMPs presumably bind to the extracellular domain of BceAB and stimulate the ATPase or GTPase activity of the transporter, triggering or enhancing the phosphorylation of TCS01 (steps 2 and 3). Consequently, the RR upregulates the operon containing the *bceAB* genes but does not regulate the *tcs01* operon (step 4). The overexpression of BceAB mediates antimicrobial peptide resistance (step 5).

In this study, we first demonstrated that TCS01 is involved in antimicrobial peptide resistance, by upregulating the expression of the ABC transporter Spr0812/Spr0813 in the presence of specific antimicrobial peptides. The ABC transporter belongs to the BceAB subfamily, whose members are known to function in tight cooperation with cognate TCS systems in Gram-positive bacteria with a low G+C content (Firmicutes) [22,53]. A distinctive feature of these BceAB-type transporters is that they are not only responsible for resistance but are also essential for signaling the presence of antimicrobial peptides to the cognate histidine kinase [45], since they carry an extracellular sensory domain of the system [37]. Consistent with the fact that mutations that prevent ATP hydrolysis abolish signal transduction by BceAB transporters [20,45], binding of antimicrobial peptides to the extracellular domain of BceAB stimulate its ATPase activity and may thus trigger/stimulate a phosphorylation cascade from HK01 to RR01 that ultimately results in the transcriptional upregulation of the *bceAB* genes (Fig 8). The degree of ATPase stimulation by the antimicrobial peptides that we revealed *in vitro* is substantial, especially since bacterial ABC transporters with large specificities such as multidrug transporters tend to be weakly stimulated by their substrates [54]. The regulation in *S*. *pneumoniae* (Fig 8) is clearly different from the homologous system in *Streptococcus mutans*, in which the *ABC* and *TCS* genes are adjacent on the genome and are both upregulated by bacitracin [55]. Consistent with our analysis of *S*. *pneumoniae* transcription levels, TCS01 expression has not been found to be affected by the presence of bacitracin or other AMPs in other studies [24,56]. This may explain why the physiological role of TCS01 has been cryptic so far, especially considering that another TCS was found to respond to bacitracin. The LiaRS stress response system (TCS03) was indeed found to be activated by cell wall perturbations and antimicrobials like vancomycin and bacitracin [56]. The abundance of LiaS was increased by two-fold in our proteomics study when the R6 Δ*tcs01* strain was treated with bacitracin. In *Bacillus subtilis*, the BceAB system respond to bacitracin directly (drug sensing), whereas the LiaRS system respond only at higher concentrations and indirectly to bacitracin action (damage sensing) [20,57]. The bacitracin-induced overexpression of the BceAB transporter but not the TCS01 system strongly suggests that the resistance is primarily mediated by the ABC transporter (Fig 5). This is in line with the observation that the heterologous expression in *Lactococcus lactis* of NsrFP, a BceAB-type transporter from *Streptococcus agalactiae*, was able to confer a 16-fold resistance against nisin [58].

The substrate specificity displayed by BceAB suggests that it confers resistance to antimicrobial peptides that minimally require the pyrophosphate moiety of the lipid carrier of cell wall synthesis, i.e. undecaprenyl pyrophosphate (C55-PP), as a docking component to exhibit antimicrobial activity. Both bacitracin and nisin bind the pyrophosphate moiety but inhibit different steps of the lipid II cycle [27,30]. While bacitracin inhibits the dephosphorylation of undecaprenyl pyrophosphate for recycling [59], nisin sequesters lipid II and uses it as a ‘docking molecule’ to form pores in a targeted and efficient manner [28,31]. The BceAB from *S*. *pneumoniae* has sequence similarities with all three BceAB-type transporters in *Bacillus subtilis*. While *B*. *subtilis* employs two separate transporters, YvcRS (renamed PsdAB) and BceAB for resistance against nisin and bacitracin, respectively [21], BceAB protects against both molecules in *S*. *pneumoniae*. It should be noticed that BceAB confers resistance to actagardin but not to ramoplanin, albeit they both bind lipid II and share a conserved backbone [60]. However, ramoplanin requires the presence of MurNAc-Ala-Glu pyrophosphate in order to bind to lipid II [60], while actagardin was proposed to interact with the MurNAc-GlcNAc pyrophosphate of Lipid II [61]. Consistent with these differences, it was observed that actagardin but not ramoplanin induces the expression of the *bceAB* operon [21]. Furthermore, we showed that ramoplanin could inhibit the ATPase activity of BceAB, which may also explain why the transporter fail to mediate resistance to this antimicrobial peptide. The exact mechanism of resistance has been highly debated in the field. A direct export mechanism was initially suggested [19,58]. While general efflux could explain the surprisingly broad range of structurally-unrelated AMPs to which BceAB systems respond, it is however unclear why these transporters only seem to protect against AMPs that target cell-wall biosynthesis, as clearly observed here with the BceAB transporter from *S*. *pneumoniae*. It was recently suggested that BceAB-type transporters are mechanotransducers that confer resistance via target protection. The C-terminal four transmembrane helices and intervening periplasmic domain of BceB are topologically similar to the mechanotransducer MacB [43]. In line with this hypothesis, it was proposed that BceAB-type transporters could transiently release lipid II cycle intermediates from the inhibitory sequestration of antimicrobial peptides [44]. In this elegant model, BceAB would recognize its substrate AMPs in complex with their respective cellular target (i.e. UPP-AMP or lipid II-AMP). The ATPase activity of the transporter would provide the necessary energy to disrupt, via conformational changes that propagate to the extracellular domain, the interaction between the AMP and cell-wall precursors. If the transporter has a lower affinity for the free AMP as compared to the AMP in complex with its cellular target, this could promote a fast release of the AMP once it is dissociated from the target. By freeing UPP or lipid II from the grasp of AMPs, BceAB could ensure that the impacted enzymes (UPP-phosphatases or peptidoglycan transglycosylases, respectively) catalyze the next step of cell wall synthesis, enabling the cycle to continue for a few rounds before the AMP can rebind to its target [44].

Because *S*. *pneumoniae* is a commensal or opportunistic pathogen, it is likely exposed to a variety of bacterial and human antimicrobial peptides present in the different environments of the human host. Thus, the discovery of the functional role of TCS01 in antimicrobial peptide resistance explains, at least partially, its implication in virulence reported by a number of independent studies [13,15,16]. In line with this, two other pneumococcal TCS implicated in virulence [14] respond to antibiotics and cell wall perturbations: CiaRH (TCS05) and WalRK (TCS02) [9]. Besides its remarkable genetic plasticity [62], *Streptococcus* also encodes an extended range of regulatory molecules, ranging from “stand-alone” transcriptional regulators, two-component systems and eukaryotic-type kinases to regulatory RNAs [1,63]. These systems largely contribute to the adaptability of the opportunistic human pathogen when it encounters various environments in the host [2]. Interestingly, a recent transcriptomic study conducted under a wide range of infection-relevant conditions identified _~_500 conditionally-expressed pneumococcal genes, including the *bceA* gene but none of the *tcs01* genes [64]. Competition among microorganisms is a key factor of successful host colonization and persistence [65]. The success of *S*. *pneumoniae* as a colonizer first requires complex interactions with the nasopharyngeal microbiota [2,66]. Commensal bacteria indeed inhabit mucosal and epidermal surfaces in humans and play an important role in defense against pathogens, including respiratory ones [67]. These bacteria can directly inhibit the growth of respiratory pathogens by not only competing for nutrients and adhesion sites but also by producing antimicrobial compounds or signals. Altogether, these competitive mechanisms preserve the niche for commensal bacteria and support the host in holding respiratory infections [67]. A prominent example for a commensal bacterium producing AMPs is *Streptococcus salivarius*, which secretes a wide range of bacteriocins that antagonizes *S*. *pneumoniae* [68–70]. Besides ribosomally encoded antimicrobials, commensal bacteria also encode non-ribosomally produced bioactive antimicrobials to compete with pathogens [71]. Successful pathogenic colonizers must also evade the host immune response. As part of the innate immunity against invasive diseases, epithelial cells and neutrophils can synthesize antimicrobial peptides, including cathelicidins and defensins, as direct killing mechanisms against bacteria [72]. Some human defensins have also been suggested to interact with Lipid II [73,74], and one of our future objectives is to investigate whether BceAB confers resistance to some of them, although preliminary work suggest that it does not promote resistance to the human neutrophil defensin HNP1 (S7 Fig). These human peptides can also act as chemokines to recruit neutrophils [75] and contribute to host immunity by assisting in maintaining the balance between protection from pathogens and tolerance to normal flora [76].

To summarize, our study showed that TCS01 is involved in the upregulation of an antimicrobial peptide resistance system that likely contributes to the overall pathogenicity and virulence of *S*. *pneumoniae*. If colonizing bacteria are resistant to the effects of local antimicrobial peptides synthesized by commensal bacterial competitors or immune cells during the inflammation process, every stage of colonization can be promoted, leading to an efficient bacterial propagation [77].

## Materials and methods

### Bioinformatic analyses

The UNIPROT database was used to obtain the amino acid sequences of the proteins of interest. The National Centre for Biotechnology Information website was used for protein BLAST searches (https://blast.ncbi.nlm.nih.gov/Blast.cgi). Primary sequence alignments and percentages of similarity were obtained by using the NPS@ servor (https://npsa-prabi.ibcp.fr/cgi-bin/npsa_automat.pl?page=/NPSA/npsa_clustalw.html) [78].

### Construction of R6 and D39 knock-out strains

All the strains used in this study are listed in Table 1. Genomic DNA was isolated from a 5 ml culture of *S*. *pneumoniae* R6 or D39 grown to optical density (OD_600nm_) 0.3 at 37°C with 5% CO_2_ in Todd-Hewitt (TH) broth (*Difco*) supplemented with 0.5% w/v yeast extract when necessary (THY). The culture was spun down at 20,000 g for 10 min and the pellet resuspended in 500 μL PBS containing lysozyme (10 μg/mL) and mutanolysine (0.5 μg/mL) and incubated at 37°C for one hour. Then the genomic DNA was extracted using the High Pure PCR Template Isolation kit (*Roche*) according to the manufacturer’s instructions. The quality and quantity of the DNA extraction was performed using NanoVue Spectrophotometer (*GE Healthcare*).

For in-frame deletion of the *bceAB* genes, a construct was created in which 869 bp of flanking DNA 5′ to the *NBD* ATG (primers NBD-F and NBD cat-R; see list of primers in S4 Table) and 848 bp of flanking DNA 3′ to the *TMD* open-reading frame (ORF) (primers cat TMD-F and TMD-R) were amplified by PCR from *S*. *pneumoniae* D39 genomic DNA and fused to the chloramphenicol resistance cassette (*CAT*, amplified from pR326 [79], with primers NBD CAT-F and CAT TMD-R) by overlap extension PCR [80]. The fragments were amplified using 2.5 μL of primers (100 μM), 1 μL of 10 mM dNTP (Thermo Scientific), 10 μL of 5 x High Fidelity buffer (Thermo Scientific) and 0.5 μL of Phusion polymerase protein (Thermo Scientific) in a 50 μL final volume. The PCR program was as follows: 98°C for 30 sec, then a 35 time repeat of 98°C for 10 sec, 48°C for 30 sec, and 72°C for 90 sec, after the last cycle at 72°C for 8 min and samples were kept at 4°C. The DNA samples were analyzed between each step by electrophoresis in 1% agarose gel and TAE buffer (Tris-acetate 40 mM pH 8, EDTA 1 mM). The fragments were purified using the QIAquick Gel Extraction Kit (Qiagen) and eluted in 25 μL of water and concentration measured using NanoDrop. Equal weight of the three fragments were mixed and submitted to PCR to be fused together. Primers used for the overlap extension PCRs were NBD-F and TMD-R.

*S*. *pneumoniae* genome was afterwards transformed by homologous recombination and allelic replacement using competence stimulating peptide (CSP-1) and standard protocols [81,82] where 10 mL of D39 or R6 were grown to OD_600nm_ 0.15 and the culture was spun down for 10 min at 3200 g. The pellet was then resuspended in 1 mL THY pH = 8 containing 0.2% (v/v) BSA and 1 mM of CaCl_2_. Then 1 μL of CSP1 (0.5 μg/mL) was added for 7 min incubation at 37°C. After addition of 20 μL of PCR product growth was carried out for 5 h. Finally, 100 μL of bacteria were spread on Columbia blood agar (Difco) plates containing 5% v/v of horse blood with chloramphenicol (4.5–10 μg/ml) in serial dilutions from undiluted bacteria to 10^−6^. Only the 10^−6^ dilution was spread for the control culture, which underwent the same treatment as transformed bacteria without any DNA being added.

For deletion of the *tcs01* genes, a construct was created as described above: 835 bp of flanking DNA 5′ to the *RR* ATG (primers RR-F and RR kana-R) and 853 bp of flanking DNA 3′ to the *HK* ORF (primers kana HK-F and HK-R) were amplified by PCR from *S*. *pneumoniae* D39 genomic DNA and fused with the kanamycin resistance marker (*KANA*, amplified from pLIM100, with primers RR kana-F and kana HK-R). The final product was 2610 bp long. PCR, purification and transformation protocols were identical to the ones described above. A concentration of 50 μg/mL of kanamycin was used for the selection.

The mutations were tested by PCR on positive clones amplifying the insert by outside test primers T1-ABC-F and T2-ABC-R for ABC deletion and T3-TCS-F and T4-TCS-R for TCS deletion. The expected sizes for the strains were WT 4804 bp Δ*bceAB* 3155 bp and WT 4628 bp Δ*tcs01* 3900 bp respectively. The constructs were then additionally verified by DNA sequencing.

### Construction of R800 mutant strains

Gene modifications (Table 1) in *S*. *pneumoniae* R800, which is streptomycin resistant, were achieved by homologous recombination, using a two-step procedure based on the biscistronic Janus cassette kan-rpsL [35]. This cassette confers the Kanamycin resistance and Streptomycin sensitivity in R800. The Janus cassette was then removed or fused to gfp by homologous recombination. The antibiotics were used at final concentration of 250 μg/mL for kanamycin and 200 μg/mL for streptomycin. Cells were growth at 37°C until OD_550nm_ = 0.1 and then incubated with the synthetic competence stimulating peptide and the DNA fragment of interest for 30 min at 37°C. Transformants were then plated onto THY-agar medium supplemented with 4% defibrinated horse blood and incubated 2 h at 37°C. The selection of transformants was made with the appropriate antibiotic (streptomycin or kanamycin). Clones were checked by DNA sequencing.

### Immunoblots

Cells were inoculated in C+Y medium [83] and grown at 37°C, without agitation and in anaerobic conditions, until at OD_550nm_ = 0.3. After centrifugation 5 min at 5,000 g, pellets were resuspended in 1/50^th^ of initial volume in TE buffer (25 mM Tris-HCl pH = 7.5, EDTA 1 mM) with DNase/RNase 6 μg/mL and protease inhibitor CLAPA 1X (Chymostatin 1 μg/mL, Pepstatin 1 μg/mL, Leupeptin 1 μg/mL, Antipaïn 1 μg/mL, Aprotinin 4 μg/mL). Cells were lysed by sonication by using Sonifier 450 Branson device. 25 μg of crude extracts were mixed with Laemmli loading buffer and heated 10 min at 95°C. Samples were loaded onto an 12.5% SDS-PAGE and migration was performed in TG-SDS buffer (Tris-HCl 25 mM, Glycine 0.192 M, SDS 0.1% (v/v)). Proteins were transferred onto a PVDF membrane, previously activated by methanol 100%, in a transfer buffer (Tris-HCl 25 mM pH = 8, Glycine 0.192 M, methanol 20% (v/v), SDS 0.04% (v/v)). The membrane was washed with TBST (Tris-HCl 100 mM pH = 8, NaCl 150 mM, Tween 0.05%) and incubated 1 h with blocking buffer (BSA 5%, TBST). After a washing step with TBST, the membrane was incubated with primary antibody during 1 h: an anti-GFP antibody at 1/10 000 (AMS Biotechnology) or anti-Enolase antibody at 1/50 000 [84], in TBST supplemented with 1% BSA. Membranes were washed three times 10 min with TBST. Goat anti-rabbit immunoglobulin HRP secondary antibody (Bio-Rad) was then used at 1/5000 in TBST supplemented with 1% BSA during 1 h. After three washing steps in TBST, immunoblots were revealed with the « SuperSignal West Pico Chemluminescent Substrate » kit of Thermo Scientific and images acquired by Fusion camera of Vilber-Lourmat.

### Source of antimicrobial peptides

The AMPs were purchased from Sigma-Aldrich (Bacitracin, nisin, and gramicidin), Adipogen (actagardin, ramoplanin, planosporicin, Microbisporicin, NAI-802, NAI-857 and colistin), Smart-Bioscience (mastoporan), Bio Basic Canada (vancomycin) and Vivitide (HNP-1).

### MIC determination

The MIC of various antimicrobial peptides for R6, R800 and D39 strains was determined by classical 2-fold broth dilution method. Cells were first grown in Todd-Hewitt medium at 37°C without agitation and in anaerobic conditions until the absorbance reaches approximatively 0.3. Complementation strains were grown with 0.5 μM ComS inducer until the appropriate OD. Cells were then diluted at final OD_600nm_ = 0.002 in 96-well plates containing 300 μL of Todd-Hewitt medium (and 2 μM ComS inducer for the complementation strains) with serial dilutions of antimicrobial peptides. Pneumococcal growth was monitored by a microplate reader (TECAN) at 37°C without agitation. The absorbance was followed at 600 nm every 15 min for 15 h.

### Susceptibility to HNP-1

Bacterial cells were routinely grown until OD_600nm_ = 0.3 in THY and 5 mL of culture were centrifuged for 10 min at 5,000 g at 4°C. The pellet was resuspended in 5 ml of sterile PBS buffer and diluted twice at 1/100. 200 μl of the final dilution was incubated for 30 min, 1 h or 2 h at 37°C, 5% CO_2_ in 96-well plate with 5, 10 or 20 μg/ml of hNP1. Next, 10 μl from the final dilution (control cells at time zero in the absence of HNP-1) and the 96-well plate (cells incubated with HNP1) were plated onto pre-cast THY-agar medium supplemented with 4% defibrinated horse blood and incubated overnight at 37°C, 5% O_2_. The next day, colonies were counted.

### Quantitative PCR

*S*. *pneumoniae* D39 WT and Δ*tcs01* strains were routinely grown to OD_600nm_ = 0.3 in THY. Then 1 μg/mL of bacitracin was added to the growth media for up to 30 min. Samples were taken at time points shown in the results section. Next, the cultures were mixed with RNA protect (Qiagen) at 1:1 ratio and incubated at room temperature for 5 min and centrifuged for 10 min at 3, 200 g at 4°C. The supernatant was decanted and the pellet was stored at -80°C. The bacteria were resuspended in 100 μL of sterile Tris-EDTA (TE), containing 15 mg/mL lysozyme (Sigma) then 20 μL of Proteinase K (20 mg/mL) (Roche) were added. The sample was vortexed 5 times for 10 sec at two-minutes intervals. Then 350 μL of lysis buffer from the Nucleospin RNA kit (Macherey-Nagel) were added to the sample with 3.5 μL of β-mercaptoethanol and 25 mg of glass beads (Sigma). The cells were lysed by continual vortexing for 5 min. The mRNA was extracted according to the manufacturer’s instructions. After elution, RNA samples were incubated with 5 μL of DNAse for 20 min at room temperature, then inactivated by heating for 70°C for 5 min. RNA concentration was measured using Nanovue (GE Healthcare). For cDNA synthesis 1 μg of RNA was generally used with the SuperScript III kit (Invitrogen) containing a reverse transcriptase, a set of primers, dNTPs, MgCl_2_ random hexamers and RNaseOUT which digests left-over RNA after the cDNA generation according to manufacturer’s instructions. Samples were then stored at -20°C. qPCR measurements were done with 2.5 μL of cDNA mixed with 1 μL (3 μM final) of forward and reverse primers, 5 μL iQ SYBR Green Supermix (BioRad) and 0.5 μL water using the CFX Connect Optical Module (BioRad) program as follows: 95°C for 3 min, then a repeat 40 times of 95°C for 10 sec, 55°C for 30 sec, and a fluorescence reading.

### Microscopy

Pneumococcal cells were grown at 37°C until OD_550nm_ = 0.2 in C+Y medium [83]. Cells were incubated with or without bacitracin 0.5 μg/mL during 30 min at 37°C. Cells were then imaged after 10 min of incubation with a Nikon TiE microscope with NIS-Elements (Nikon) through a 100X 1.45 numerical aperture objective. For membrane staining, cells were loaded on an agarose pad containing 10 μg/mL of FM4-64. The microscope was fitted with an ORCA-Flash4.0 V2 digital CMOS camera (Hamamatsu Photonics) for image capturing. Images were analyzed using ImageJ (http://rsb.info.nih.gov/ij/) and the plugin MicrobeJ to generate violin plots [85].

### Proteomic analysis of the R6 and R800 strains

In experiments 1 and 2, we analyzed the R6 WT and R6 *Δtcs01* strains treated or not with 1 μg/mL of bacitracin. In experiment 3, we analyzed the R800-Δ*hk01*-P_*comX*_-*rr01-hk01* strain treated with either 1 μg/mL or 32 μg/mL of bacitracin.

#### Sample preparation

Strains were grown in 50 mL of THY medium at 37°C without agitation and in anaerobic conditions until the absorbance reaches approximatively 0.3. After the addition of bacitracin (final concentrations of 1 or 32 μg/mL), the bacterial cultures were incubated for 45 min at 37°C with a slight mechanical agitation (60 rpm). Then, the cell density was adjusted to an OD_600nm_ = 0.8 in 10 mL. After centrifugation at 4,000 x g for 10 min at 4°C, the pellets were resuspended with 1 mL of cold Tris-HCl 50 mM pH 7.5 and centrifuged at 10,000 x g, for 5 min at 4°C. The pellets were harvested and treated in the same conditions than previously. The contents were transferred into Eppendorf low-binding tubes. After a last centrifugation at 10,000 x g for 5 min at 4°C, the bacterial pellets were stored at -80°C. After thawing the pellets, 300 μL of lysis buffer were added (Tris-HCl 10 mM pH 8, EDTA 1 mM and antiproteases cocktail). Cells were lysed at 4°C using Bioruptor by 2 cycles of 15 min (30 sec of ultrasounds and 30 sec of rest). Each tube was supplemented with 700 μL of lysis buffer and 100 μL of foscholine 12 10% (final concentration at 0.9%) to solubilize membrane proteins [48]. The samples were incubated for 1.5 h at 4°C on a wheel rotating at 30 rpm. The bacterial lysates were centrifugated at 150,000 x g for 1h at 4°C. The supernatants containing soluble and solubilized proteins were harvested and transferred in Eppendorf low binding tubes. The proteins were quantified by using BCA assay kit (Thermo Scientific) before sending the samples to mass spectrometry analysis.

#### In solution digestion and TMT labeling

To compare the proteins abundance between the different conditions/samples, we used a multiplexed quantitation method after labeling by isobaric Tandem Mass Tags (TMT) [42]. Protein samples were in-solution digested, TMT-labeled, and fractionated for experiments 1 and 3 according to the published protocol [86]. For experiment 2, the sample was directly desalted after TMT labeling using spin column C18 (Thermo Scientific).

#### TMT experiment design

We simultaneously compared 6 TMT samples within the same experiment. In the experiment 1 we analyzed one sixplex containing R6 WT, R6 WT+ Bacitracin 1, R6 WT+ Bacitracin 2, R6 ΔTCS, R6 ΔTCS+ Bacitracin 1, R6 ΔTCS+ Bacitracin 2 labeled with reporter ions 126, 127, 128, 129, 130 and 131, respectively; and experiment 2 is a second sixplex containing R6 WT1, R6 WT2, R6 WT+ Bacitracin, R6 ΔTCS1, R6 ΔTCS2, R6 ΔTCS+ Bacitracin labeled with reporter ions 126, 127, 128, 129, 130 and 131, respectively. The calculated ratios shown in Figs 5 and S5 are: experiment 1: R6 WT+ Bacitracin (1/2)/R6WT and R6 WT+ Bacitracin (1/2)/ R6 ΔTCS+ Bacitracin (1/2); experiment 2: R6 WT+ Bacitracin /R6WT (1/2) and R6 WT+ Bacitracin/ R6 ΔTCS+ Bacitracin. The calculated ratios shown in S6 Fig were calculated from the average of two biological replicates: Δ*hk01*-P_*comX*_-*rr01-hk01* strain treated with 32 versus 1 μg/mL of bacitracin.

#### LC-MS/MS analysis

Samples were analyzed in triplicate using an Ultimate 3000 nano-RSLC (Thermo Scientific, San Jose California) coupled on line with a Q Exactive HF mass spectrometer via a nano-electrospray ionization source (Thermo Scientific, San Jose California).

The fractions of experiment 1 were analysed by nanoLC-MS/MS with the same experimental conditions already described in Nolivos et al. [86], excepted for preconcentration conditions: fractions were injected onto a C18 Acclaim PepMap100 trap-column 300 μm ID x 5 mm, 5 μm, 100Å, (Thermo Scientific) for 3 min at 20 μL/min with 2% Acetonitrile MS grade (ACN, Sigma Aldrich), 0.05% trifluoroacetic acid (TFA) in H_2_O.

For experiment 2 sample, 500 ng of labeled peptide mixture were injected, loaded and preconcentrated on trap-column as similar as experiment 1 and then separated on a C18 Acclaim Pepmap100 nano-column, 75 cm x 75 μm i.d, 2 μm, 100 Å (Thermo Scientific) with a 160 min linear gradient from 3.2% to 40% buffer B (A: 0.1% Formic Acid (FA) in H_2_O, B: 0.1% FA in ACN) and then from 40 to 90% of B in 22 min, hold for 5 min and returned to the initial conditions in 1 min for 19 min with a flow rate of 300 nL/min and the oven temperature set at 50°C. Labeled peptides were analyzed with TOP15 HCD DDA method in the MS scan 375–1800 Th. The resolution of the survey scan was 120,000 at m/z 200 Th and for MS/MS scan the resolution was set to 30,000 at m/z 200 Th for experiment 1 and 45000 at m/z 200 Th for experiments 2 and 3. Parameters for acquiring HCD MS/MS spectra were as follows; collision energy = 32 for experiment 1 or CE = 33 for experiment 2 and 3 and an isolation width of 1.2 m/z. The precursors with unknown charge state, charge state of 1 and 8 or greater than 8 were excluded. Peptides selected for MS/MS acquisition were then placed on an exclusion list for 30 s using the dynamic exclusion mode to limit duplicate spectra.

#### Data analysis

Proteins were identified by database searching using SequestHT with Proteome Discoverer 2.2 software (Thermo Scientific) against the uniprot *Streptococcus pneumoniae* R6 database (2019–01 release, 2822 sequences). Precursor mass tolerance was set at 10 ppm and fragment mass tolerance was set at 0.02 Da, and up to 2 missed cleavages were allowed. Oxidation (M), acetylation (Protein N-terminus) and TMT labeled peptides in primary amino groups (+229.163 Da Lys and N-ter) were set as variable modification, and Carbamidomethylation (C) as fixed modification. Peptides and proteins were filtered with a false discovery rate (FDR) at 1% using percolator and proteins were identified with 1 unique peptide in rank 1. Protein quantitation was performed with reporter ions quantifier node in Proteome Discoverer 2.2 software with integration tolerance of 20 ppm, peptide and protein quantitation based on pairwise ratios and ANOVA hypothesis test. Proteins functional annotation was performed with GO (Gene ontology). The mass spectrometry proteomics data have been deposited to the Center for Computational Mass Spectrometry repository (University of California, San Diego) via the MassIVE tool with the dataset identifier MassIVE MSV000089094. Furthermore, the analyzed data are available in S1 Data (see the outline sheet to find the necessary information).

### Cloning of bceAB genes in the pRSF-Duet-1 expression plasmid

First, the genes encoding the NBD (*spd_0804*) and the TMD (*spd_0805*) of the transporter were PCR-amplified from *S*. *pneumoniae* D39 genome by using the Pfu-polymerase (Promega) and the primers BceA-1 and BceB-1. A second PCR was performed using the primers BceA-2 and BceB-2 to enable the insertion of a polyhistidine tag on the *N*-terminus of the NBD and the *C*-terminus of the TMD respectively, using the first PCR fragment as a template. The pRSF-Duet1 plasmid and the second his-NBD-TMD-his fragment were both sequentially digested by first NcoI restriction enzyme (*New-England Biolabs*) and by EcoRI restriction enzyme (*New-England Biolabs*) according to the manufacturer’s instructions. Next, the fragment and the plasmid were ligated using Rapid DNA ligation kit (*Roche*). The ligation reaction was used to transform XL10Gold competent bacteria (*Invitrogen*). Positive clones were selected by restriction digest and verified by sequencing.

### Site-directed mutagenesis

BceAB-K48A, BceAB-E170Q and RR01-D52E mutations were engineered by using the QuikChange Lightning Site-Directed Mutagenesis Kit from Agilent.

### Cloning of the response regulator (RR) in the pET-Duet1 expression plasmid

The RR gene (*spd_1446*) was amplified from *S*. *pneumoniae* D39 genome using primers Fwd RR01 and Rev RR01 allowing the insertion of a polyhistidine tag at the *N*-terminus of the RR protein. The pET-Duet1 plasmid and the RR fragment were both sequentially digested by NdeI and XhoI restriction enzymes according to the manufacturer’s instructions. The PCR fragment and the plasmid were then ligated. The ligation reaction was used to transform TOP10 competent bacteria. Positive clones were selected by restriction digest and verified by DNA sequencing.

### Overexpression and purification of the response regulator

*E*. *coli* BL21(DE3) cells were transformed with the plasmid pET-Duet1-RR-D52E and selected on LB-agar plates containing 100 μg/mL ampicillin. After an overnight preculture at 37°C in LB supplemented with ampicillin, cells were diluted in 1L of culture at OD_600nm_ = 0.1 and grown at 37°C, 180 rpm. At OD_600nm_ of 0.6, protein expression was induced by adding IPTG to a final concentration of 1 mM. Induction was performed overnight at 20°C and cells were then harvested by centrifugation at 6,000 g for 20 min. The pellet was frozen at -80°C until further use. The cell pellet was resuspended in buffer A (30 mL of 20 mM Tris-HCl pH 8, NaCl 300 mM and glycerol 10%) supplemented with 50 μg/mL of DNAse I and one tablet of cOmplete Protease Inhibitor Cocktail (Roche). Cells were broken by sonication (4 cycles of 3 min). Unbroken cells and debris were removed by a centrifugation at 15,000g for 20 min at 4°C. The supernatant was purified by an affinity step using HisTrap HP 1 mL column (Cytiva) equilibrated in buffer A connected to an NGC purifier (Biorad). The resin was then washed with the same buffer containing 50 mM imidazole. The RR was then eluted with 135 mM imidazole and dialyzed overnight in buffer A.

### Electrophoretic mobility shift assays (EMSA)

These assays were conducted according to the previously described strategy [41]. Three PCR products were amplified from *S*. *pneumoniae* R6 genomic DNA. A 557-bp region harbouring the promoter region of *bceAB* genes was amplified, from position -242 to +335 relative to the ATG of the *NBD* gene (primers pBceAB-F and pBceAB-R). A 379-bp fragment located within the *NBD* gene sequence from position +238 to +617 (primers BceA-F and BceA-R) and a 250-bp sequence in the promoter region of the pneumolysin *ply* gene, from position -115 to +135 (primers Ply-F and Ply-R), were amplified as negative controls. All fragments were amplified by PCR using the Q5 High-Fidelity DNA polymerase (NEB) and purified with the NucleoSpin Gel and PCR Clean up kit (Macherey-Nagel). For the EMSA assays, 100 ng of PCR products (target DNA and controls) were mixed with 2.5 μg of the purified RR01-D52E mutant (corresponding to > 140 molar excess), in 20 μl of Tris-HCl 20 mM pH 8.3, NaCl 50 mM, Glycerol 10%. The protein-DNA mixture was incubated at room temperature for 30 min and then separated on a 7.5% native polyacrylamide gel at 170 V using a precooled TBE running buffer (Tris base 44 mM, boric acid 44 mM, 1 mM EDTA). The gels were then stained with GelRed at room temperature during 30 min and photographed.

### Overexpression and purification of BceAB

*E*. *coli* BL21(DE3) cells were transformed with pRSF-Duet1-BceAB WT, K48A or E170Q and selected on LB-agar plates containing 50 μg/mL kanamycin. One colony was inoculated into in 2L-baffled flasks containing 500 mL of 2YT medium and 50 μg/mL kanamycin and grown at 37°C, 180 rpm to OD_600_ of 0.6. Protein expression was induced by adding IPTG to a final concentration of 1 mM. Induction was performed overnight at 25°C and cells were harvested by centrifugation at 7,000 g for 20 min.

Cells were resuspended in buffer A (HEPES-NaOH 100 mM pH 8.0, NaCl 150 mM) and lysed by passaging them three times through a cell disruptor (Microfluidizer, Microfluidics) at 18,000 psi (or 1,24 x 108 Pa). Undisrupted cells and cell debris were removed by centrifugation at 15,000 x g for 30 min at 4°C. Membranes were harvested from the supernatant after ultracentrifugation at 150,000 x g for 1 h at 4°C. Membranes were resuspended in 20 mL of buffer A and centrifuged once again at 150,000 x g for 1 h at 4°C. Membrane pellets were homogenized in buffer HEPES-NaOH 100 mM pH 8.0, NaCl 150 mM and 20% glycerol, and stored at -80°C.

For the purification of BceAB, membranes from 0,5 L cell culture were diluted in buffer B (100 mM d’HEPES-NaOH pH 8, 150 mM NaCl, 10% glycerol) to a protein concentration of 4 mg/mL and solubilized with 0.5% (w/v) LMNG for 2 h at 4°C. After ultracentrifugation at 150,000 x g for 1 h at 4°C, the supernatant containing solubilized proteins was diluted four times using buffer B and loaded on Ni^2+^-charged immobilized metal-ion affinity chromatography (IMAC) column (1 mL HisTrap HP, Cytiva). The column was washed with 10 mL of buffer B containing 0.005% (w/v) LMNG and 20 mM of imidazole. The column was further washed with the same buffer until OD_280nm_ reached basal levels. BceAB was eluted by buffer B containing 0.005% (w/v) LMNG and an imidazole gradient from 20 to 500 mM of imidazole. Purified BceAB was dialyzed into buffer B containing 0.005% (w/v) LMNG and stored at -80°C.

### Reconstitution of BceAB in liposomes

The proteoliposomes were prepared as previously described for the transporter BmrA [50]. Proteoliposomes were loaded on sucrose gradient with different layers of 400 μL of 50 mM HEPES-KOH pH 8.0 buffer containing 30, 20, 15 or 10% of sucrose. One mL of proteoliposomes was loaded on the top of the sucrose gradient and ultra-centrifuged at 200,000 x g for 12 h at 4°C. Proteoliposomes were typically collected from the 15% layer and were loaded on SDS-PAGE to analyze and quantify the presence of BceAB by using a standard-curve of purified BceAB and ImageJ.

### ATPases and GTPases assays

These activities were performed as previously described [48].

## Supporting information

S1 Table Blastp search results in *Bacillus subtilis* 168 using the HK01 or RR01 from *Streptococcus pneumoniae* D39 or R6 as query. The sequence of HK01 and RR01 are identical in D39 or R6 strains.
(DOCX)

S2 Table Blastp search results in *Streptococcus pneumoniae* (R6 or D39) using the transmembrane domains YvcS, YxdM and BceB from *Bacillus subtilis* as queries. It should be noted that only one protein was found when using each query.
(DOCX)

S3 Table Homologous protein sequences in *S*. *pneumoniae* and *B*. *subtilis*. For *Streptococcus pneumoniae*, the protein nomenclatures indicated in the table are derived from the non-pathogenic R6 and pathogenic D39 strains, respectively. Of note, the protein sequences of interest are identical in R6, D39 and TIGR4 strains. ^a^Indicates the percentage of identity between the proteins of the two organisms. ^b^Indicates the percentage of identity + strong similarity between the proteins of the two organisms obtained by ClustalW algorithm. For instance, YvcQ has 26% identity and 50% identity + strong similarity with SPD_1445.
(DOCX)

S4 Table Primers used in this study. For the construction of the strains, the position of the primers is given upstream (-) or downstream (+) from the first ATG of the NBD and HK. Sequences displayed in non-capital letters hybridize to the kanamycin or chloramphenicol resistance genes. For the qPCR and EMSA, the position of the primers is indicated according to the start codon of the gene of interest. For the cloning, underscored sequences belong to the genes of interest.
(DOCX)

S1 Fig Predicted topology of the TMD from the BceAB type ABC transporters. The server http://www.cbs.dtu.dk/services/TMHMM/ was used for this representation. Please note the presence of an extracellular domain of about 200 residues (ECD) between the transmembrane helices 7 and 8 and characteristic of the BceAB subfamily of ABC transporters.
(TIF)

S2 Fig Genetic context of the *bceAB* (panel A) and *tcs01* genes (panel B). The genes of interest are highlighted in black boxes. This figure was prepared from data obtained in biocyc.org.
(TIF)

S3 Fig Structures and targets of the different AMPs for which SpABC and SpTCS confer a resistance to. Only planosporicin, microbisporicin and NAI-857 are structurally related.
(TIF)

S4 Fig Heat maps representing the localization patterns of BceA-GFP during the cell cycle. Cellular membranes were stained with 10 μg/mL of FM4-64 (top panel) and BceA-GFP fluorescence is shown in green (middle panel). The overlay is presented at the bottom panel, where the yellow color indicates the localization of BceA-GFP at the membrane. 2166 cells were analyzed in this representative experiment of a triplicate.
(TIF)

S5 Fig Proteomic analysis of wild-type and Δ*tcs01* R6 strains with or without bacitracin treatment. Data correspond to one biological replicate (experiment 1, see Material and Methods). **A**, volcano plot showing proteins differentially expressed in the wild-type strain upon bacitracin (Bac) treatment (1 μg/ml for 45 min). **B**, volcano plot showing proteins differentially expressed in wild-type strain as compared to the ΔTCS in the presence of bacitracin. In this experiment, the response regulator of RR01 was detected in the R6 WT + bacitracin sample and was artificially found overexpressed here due to the fact that it is deleted in the R6 Δ*tcs01* strain. Proteins are significantly overrepresented when Log2 (fold change) > 1 and–Log10 (P-value) > 1.3. Proteins are significantly underrepresented when Log2 (fold change) < -1 and–Log10 (P-value) > 1.3.
(TIF)

S6 Fig Proteomic analysis of the R800-Δ*hk01*-P_*comX*_-*hk01* strain treated with bacitracin. Volcano plot showing proteins differentially expressed in the R800-Δ*hk01*-P_*comX*_-*hk01* strain upon differential bacitracin treatment (32 μg/ml vs 1 μg/ml for 45 min). Data correspond to the average of two biological replicates. Proteins are significantly overrepresented when Log2 (fold change) > 1 and–Log10 (P-value) > 1.3. Proteins are significantly underrepresented when Log2 (fold change) < -1 and–Log10 (P-value) > 1.3.
(TIF)

S7 Fig Sensitivity of the WT and mutant R6 strains to the human neutrophil peptide 1 (HNP1). **A**, sensitivity of the WT and Δ*tcs01* strains to various concentrations of HNP1 over time. **B**, sensitivity of the WT and Δ*bceAB* strains to various concentrations of HNP1 over time. Data counts are the average of triplicates and were normalized according to the control cells (Ctl), at time zero in the absence of HNP1.
(TIF)

S1 Data Proteomics data set. (XLSX)
