# Supplementary material for: Identification of a two-component regulatory system involved in antimicrobial peptide resistance in Streptococcus pneumoniae
Source: PLoS Pathog. 2022 Apr 8;18(4):e1010458. doi: 10.1371/journal.ppat.1010458 (PMC9020739; doi:10.1371/journal.ppat.1010458)
Supplement: S1 Table — The sequence of HK01 and RR01 are identical in D39 or R6 strains. (DOCX) [file ppat.1010458.s001.docx]

| Query : **HK01** from *S. pneumoniae* | | | | | | |
| --- | --- | --- | --- | --- | --- | --- |
| *Bacillus subtilis* proteins | Max score | Total score | Query cover | E-value | % identity | Uniprot code |
| Sensor histidine kinase **YxdK** | 140 | 140 | 70% | 4^-40^ | 34.4 | P42422 |
| Sensor histidine kinase **BceS** | 140 | 140 | 95% | 4^-40^ | 28.7 | O35044 |
| Sensor histidine kinase **YvcQ** | 127 | 127 | 63% | 6^-35^ | 33.3 | O06979 |
| Sensor histidine kinase **WalK** | 67.8 | 67.8 | 33% | 2^-13^ | 36.8 | Q45614 |
| Alkaline phosphatase synthesis sensor protein **PhoR** | 54.3 | 54.3 | 60% | 2^-9^ | 24.9 | P23545 |
| Query : **RR01** from *S. pneumoniae* | | | | | | |
| *Bacillus subtilis* proteins | Max score | Total score | Query cover | E-value | % identity | Uniprot code |
| Sensory transduction protein **BceR** | 199 | 199 | 99% | 2^-65^ | 44.2 | O34951 |
| Transcriptional regulatory protein **YxdJ** | 199 | 199 | 99% | 3^-65^ | 42.5 | P42421 |
| Uncharacterized transcriptional regulatory protein **YvcP** | 186 | 186 | 97% | 3^-60^ | 39.6 | O06978 |
| Uncharacterized transcriptional regulatory protein **YclJ** | 138 | 138 | 96% | 1^-41^ | 34.7 | P94413 |
| Transcriptional regulatory protein **WalR** | 136 | 135 | 99% | 1^-40^ | 32.3 | P37478 |

**S1 Table. Blastp search results in *Bacillus subtilis* 168 using the HK01 or RR01 from *Streptococcus pneumoniae* D39 or R6 as query.** The sequence of HK01 and RR01 are identical in D39 or R6 strains.
