# Supplementary material for: Identification of a two-component regulatory system involved in antimicrobial peptide resistance in Streptococcus pneumoniae
Source: PLoS Pathog. 2022 Apr 8;18(4):e1010458. doi: 10.1371/journal.ppat.1010458 (PMC9020739; doi:10.1371/journal.ppat.1010458)
Supplement: S2 Table — It should be noted that only one protein was found when using each query. (DOCX) [file ppat.1010458.s002.docx]

|  | Max score | Total score | Query cover | E-value | % identity | Uniprot code |
| --- | --- | --- | --- | --- | --- | --- |
| Query : YvcS  **spr0813/SPD_0805** | 137 | 137 | 98% | 4^-35^ | 22.9 | A0A0H2ZPF3 |
| Query : YxdM  **spr0813/SPD_0805** | 110 | 110 | 98% | 1^-26^ | 24.7 | A0A0H2ZPF3 |
| Query : BceB  **spr0813/SPD_0805** | 122 | 122 | 48% | 2^-30^ | 27.4 | A0A0H2ZPF3 |

**S2 Table. Blastp search results in *Streptococcus pneumoniae* (R6 or D39) using the transmembrane domains YvcS, YxdM and BceB from *Bacillus subtilis* as queries.** It should be noted that only one protein was found when using each query.
