# Supplementary material for: Identification of a two-component regulatory system involved in antimicrobial peptide resistance in Streptococcus pneumoniae
Source: PLoS Pathog. 2022 Apr 8;18(4):e1010458. doi: 10.1371/journal.ppat.1010458 (PMC9020739; doi:10.1371/journal.ppat.1010458)
Supplement: S3 Table — For Streptococcus pneumoniae, the protein nomenclatures indicated in the table are derived from the non-pathogenic R6 and pathogenic D39 strains, respectively. Of note, the protein sequences of interest are identical in R6, D39 and TIGR4 strains. aIndicates the percentage of identity between the proteins of the two organisms. bIndicates the percentage of identity + strong similarity between the proteins of the two organisms obtained by ClustalW algorithm. For instance, YvcQ has 26% identity and 50% identity + strong similarity with SPD_1445. (DOCX) [file ppat.1010458.s003.docx]

|  | Two-component system (TCS) | | ABC transporter | |
| --- | --- | --- | --- | --- |
| Proteins in *S. pneumoniae* R6/D39 strains | Spr1473/SPD_1445 (HK) | Spr1474/SPD_1446 (RR) | Spr0812/SPD_0804 (NBD) | Spr0813/SPD_0805 (TMD) |
| Proteins in *B. subtilis 168* | YvcQ (26^a^/50^b^)  YxdK (27^a^/56^b^)  BceS (27^a^/53^b^) | YvcP (38^a^/67^b^)  YxdJ (42^a^/70^b^)  BceR (43^a^/68^b^) | YvcR (46^a^/71^b^)  YxdL (44^a^/72^b^)  BceA (47^a^/74^b^) | YvcS (21^a^/52^b^)  YxdM (20^a^/46^b^)  BceB (21^a^/51^b^) |

**S3 Table. Homologous protein sequences in *S. pneumoniae* and *B. subtilis***. For *Streptococcus pneumoniae*, the protein nomenclatures indicated in the table are derived from the non-pathogenic R6 and pathogenic D39 strains, respectively. Of note, the protein sequences of interest are identical in R6, D39 and TIGR4 strains. ^a^Indicates the percentage of identity between the proteins of the two organisms. ^b^Indicates the percentage of identity + strong similarity between the proteins of the two organisms obtained by ClustalW algorithm [1]. For instance, YvcQ has 26% identity and 50% identity + strong similarity with SPD_1445.

**Reference**

1. Thompson JD, Higgins DG, Gibson TJ. CLUSTAL W: improving the sensitivity of progressive multiple sequence alignment through sequence weighting, position-specific gap penalties and weight matrix choice. Nucleic Acids Res. 1994;22(22):4673-80. doi: 10.1093/nar/22.22.4673. PubMed PMID: 7984417; PubMed Central PMCID: PMCPMC308517.
