# Supplementary material for: Identification of a two-component regulatory system involved in antimicrobial peptide resistance in Streptococcus pneumoniae
Source: PLoS Pathog. 2022 Apr 8;18(4):e1010458. doi: 10.1371/journal.ppat.1010458 (PMC9020739; doi:10.1371/journal.ppat.1010458)
Supplement: S4 Table — For the construction of the strains, the position of the primers is given upstream (-) or downstream (+) from the first ATG of the NBD and HK. Sequences displayed in non-capital letters hybridize to the kanamycin or chloramphenicol resistance genes. For the qPCR and EMSA, the position of the primers is indicated according to the start codon of the gene of interest. For the cloning, underscored sequences belong to the genes of interest. (DOCX) [file ppat.1010458.s004.docx]

| **Primer name** | **Primer sequences** | **Primer**  **position** |
| --- | --- | --- |
| ***Construction of the strains*** | | |
| NBD-F | GCTGTAATTTAGTCGGCAATG | -848 |
| NBD CAT-R | tcaaacaaattttcatcaagcttGAATCTCCTTTCTTAATATCCC | -24 |
| NBD CAT-F | GGGATATTAAGAAAGGAGATTCaagcttgatgaaaatttgtttga | -24 |
| CAT TMD-R | CATTTGGACAATCTTACGATAACtctagaactagtggatcccccgg | +1963 |
| CAT TMD-F | ccgggggatccactagttctagaGTTATCGTAAGATTGTCCAAATG | +1963 |
| TMD-R | GCTTGGACTACAAGTCACC | +2768 |
| T1-ABC-F | CACGTAAACGCAAAGAAGC | -217 |
| T2-ABC-R | CACAAGATTCTTTCCATCAC | +5006 |
| RR-F | GGAATTTCCAGCATCATACC | - 850 |
| RR KANA-R | cattaaaaatcaaacggatccCATGCTAGATGGTCTGAAAC | -56 |
| RR KANA-F | GTTTCAGACCATCTAGCATGggatccgtttgatttttaatg | -56 |
| KANA HK-R | CACTGTCGTTCCTTTTCCgcgtctagaaagactgag | +936 |
| KANA HK-F | ctcagtctttctagacgcGGAAAAGGAACGACAGTG | +936 |
| HK-R | CTTCAACTTGACTGACTACC | +1712 |
| T3-TCS-F | CTGTTCGTGAATTCGAATCTG | -26 |
| T4-TCS-R | CGAAGTAGAGCTGAAGTTC | +4656 |
| Spr0812-aval (+)**12-amont (+)** | GGAACTAGAATAGGACACCTC | -688 |
| Spr0812-aval (-) | GCAAGATAATAGCCAATTCC | +1467 |
| Kan-rpsL (+) | CCGTTTGATTTTTAATGGATAATG |  |
| Kan-rpsL (-) | AGAGACCTGGGCCCCTTTCC |  |
| Δ*spr0812*::*kan-rpsL* (+)  (complementary to *kan-rpsL* (-)) | GGAAAGGGGCCCAGGTCTCTGCAAGCGAGGTGAATTAGTATG | +799 |
| Δ*spr0812*::*kan-rpsL* (-)  (complementary to *kan-rpsL* (+)) | CATTATCCATTAAAAATCAAACGGCCCAGCTGAACGCGCTCAATTTC | -1 |
| *spr0812-kan-rpsL* (+)  (complementary to kan-rpsL (-)) | GGAAAGGGGCCCAGGTCTCTGCAAGCGAGGTGAATTAGTATGTTTCG | +799 |
| *spr0812-kan-rpsL* (-)  (complementary to *kan-rpsL* (+)) | CATTATCCATTAAAAATCAAACGGATTCACCTCGCTTGCCATGACAGTCAAGG | +2184 |
| Linker-*gfp* (+)  (linker) | AAACTAGACATCGAGTTCCTGCAGATGATTTCTAAAGGTGAAGAATTG |  |
| *gfp* (-) | TTATTTATACAATTCATCCATACC |  |
| *spr0812-gfp* (+)  (complementary to *gfp* (-)) | GGTATGGATGAATTGTATAAATAAGCAAGCGAGGTGAATTAGTATG | +799 |
| *spr0812-gfp* (-)  (complementary to linker-*gfp* (+)) | CTGCAGGAACTCGATGTCTAGTTTATTCACCTCGCTTGCCATGAC | +813 |
| Spr1473-amont (+) | GATGATCAGGTCATTCGTC | -646 |
| *spr1473-aval* (-) | CATAAGTACTTTGATAACGATG | +1677 |
| Δ*spr1473*::*kan-rpsL* (+)  (complementary to *kan-rpsL* (-)) | GGAAAGGGGCCCAGGTCTCTAAATAGATGAGAAAAAGCCTCCAG | + 976 |
| Δ*spr1473*::*kan-rpsL* (-)  (complementary to *kan-rpsL* (+)) | CATTATCCATTAAAAATCAAACGG**TTACTCAAGGACTAAGTTCACTTGAGC** | + 8 |
| Δ*spr1473* (+) | AAATAGATGAGAAAAAGCCTCCAGATTG | +976 |
| Δ*spr1473* (-)  (complementary to Δ*spr1473* (+)) | CCAATCTGGAGGCTTTTTCTCATCTATTTTCAAGCATGCTTCAATCCGTACCCTATTC | + 1003 |
| *cpsN* (+) | CATCGGAACCTATACTCTTTTAG | +308 |
| *cpsN-O* (-) | TTTCTAATATGTAACTCTTCCCAAT | +1106 |
| P*_comX_* (+) | TGAACCTCCAATAATAAATATAAAT | 0 |
| *cpsO* (-) | ATAACAAATCCAGTAGCTTTGG | +800 |
| P*_comX_*-*rr01-hk01* (+)  (complementary to P*_comX_* (+)) | ATTTATATTTATTATTGGAGGTTCAATGCACAAGATTTTATTAATAGAAGATGATC | 0 |
| P*_comX_*-*rr01-hk01* (-) (complementary to *cpsN-O* (-)) | AATTGGGAAGAGTTACATATTAGAAATTACTCAAGGACTAAGTTCACTTGAG | +1644 |
| *spr0813*-down (-) | CCAAGATAGGGAACTAGCCAAAAAATC | +2451 |
| Δ*spr0812-spr0813::kan-rpsL* (-) (complementary to *kan-rpsL* (+)) | CATTATCCATTAAAAATCAAACGGCCCAGCTGAACGCGCTCAATTTC | 0 |
| Δ*spr0812-spr0813::kan-rpsL* (+) (complementary to *kan-rpsL* (-)) | AGGAAAGGGGCCCAGGTCTCTAAAAAGATACCTCGACTTCAAAATCG | +2805 |
| Δ*spr0812-spr0813* (-) | CCCAGCTGAACGCGCTCAATTTC | 0 |
| Δ*spr0812-spr0813* (+) (complementary to Δ*spr0812-spr0813* (-)) | GAAATTGAGCGCGTTCAGCTGGGAAAAAGATACCTCGACTTCAAAATCG | + 2805 |
| ***qPCR*** | | |
| NBD-fwd | CAGGGCAACCAAGTAGAAGC | +52 |
| NBD-rev | CGGTGTCAGTTCCATTCAAG | +220 |
| TMD-fwd | TGCCAGTGAAGCAGAACAAC | +1464 |
| TMD-rev | CCGATAAAGAAGACACCACCA | +1625 |
| HK-fwd | GGAGAGAGGGAAGCCAAGTC | +226 |
| HK-rev | GCAATGGGGGTCTTTATCTG | +386 |
| RR-fwd | GTTTGGGCGTGATGAGAGTT | +363 |
| RR-rev | CGAGCCACATTGACAGAGAG | +593 |
| PatB-fwd | GCAACCCACATTCACGACTA | +1381 |
| PatB-rev | CCTGCTACAACCACCTCCAT | +1601 |
| ***Cloning*** | | |
| BceA-1 | ggACACTTTTAGATGTAAAACAC |  |
| BceB-1 | ctaCATTTGGACAATCTTACG |  |
| BceA-2 | GATATACCATGGGGCATCATCATCATCATCATCATACACTTTTAGATGTAAAACAC |  |
| BceB-2 | GAGCTCGAATTCCTAATGATGATGATGATGATGATGATGCATTTGGACAATCTTACG |  |
| Fwd RR01 | gatatacatATGCATCATCATCATCATCATCATCATATTGAAGGCCGCCACAAGATTTTATTAATAG |  |
| Rev RR01 | cagactcgagTCAAGCATGCTTCAATCCG |  |
| ***EMSA*** | | |
| pBceAB-F | ATATTTCACCCAATCTTACAAAAATGTAAGATTAAAGTCT | -242 |
| pBceAB-R | AGCGGAAGCAAGATATTGTC | +335 |
| BceA-F | AGGCCTCTAGCTTCCGTCGTGAAAAGT | +238 |
| BceA-R | GCTGTTGAGTGGGTTACCATGAGAATGGTT | +617 |
| Ply-F | GTCTCAGAAGAATTGTACGAGGAAATTTTGGATCACTTAG | -115 |
| Ply-R | CTAAGTGATCCAAAATTTCCTCGTACAATTCTTCTGAGAC | +135 |

**S4 Table. Primers used in this study**. For the construction of the strains, the position of the primers is given upstream (-) or downstream (+) from the first ATG of the NBD and HK. Sequences displayed in non-capital letters hybridize to the kanamycin or chloramphenicol resistance genes. For the qPCR and EMSA, the position of the primers is indicated according to the start codon of the gene of interest. For the cloning, underscored sequences belong to the genes of interest.
