# Supplementary figures and images for: Identification of a two-component regulatory system involved in antimicrobial peptide resistance in Streptococcus pneumoniae
Source: PLoS Pathog. 2022 Apr 8;18(4):e1010458. doi: 10.1371/journal.ppat.1010458 (PMC9020739; doi:10.1371/journal.ppat.1010458)

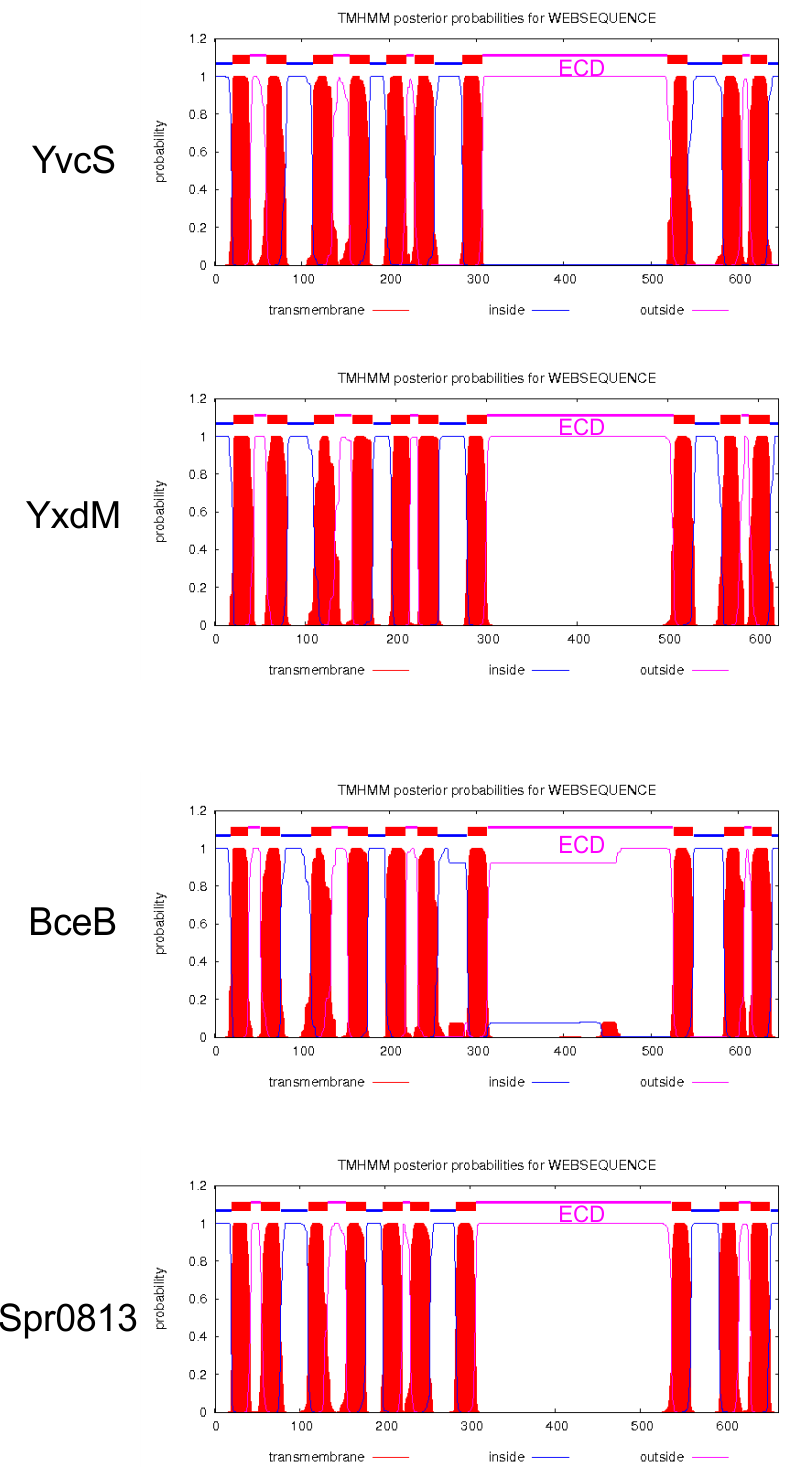

Supplement: S1 Fig — The server http://www.cbs.dtu.dk/services/TMHMM/ was used for this representation. Please note the presence of an extracellular domain of about 200 residues (ECD) between the transmembrane helices 7 and 8 and characteristic of the BceAB subfamily of ABC transporters. (TIF) [file ppat.1010458.s005.tif]

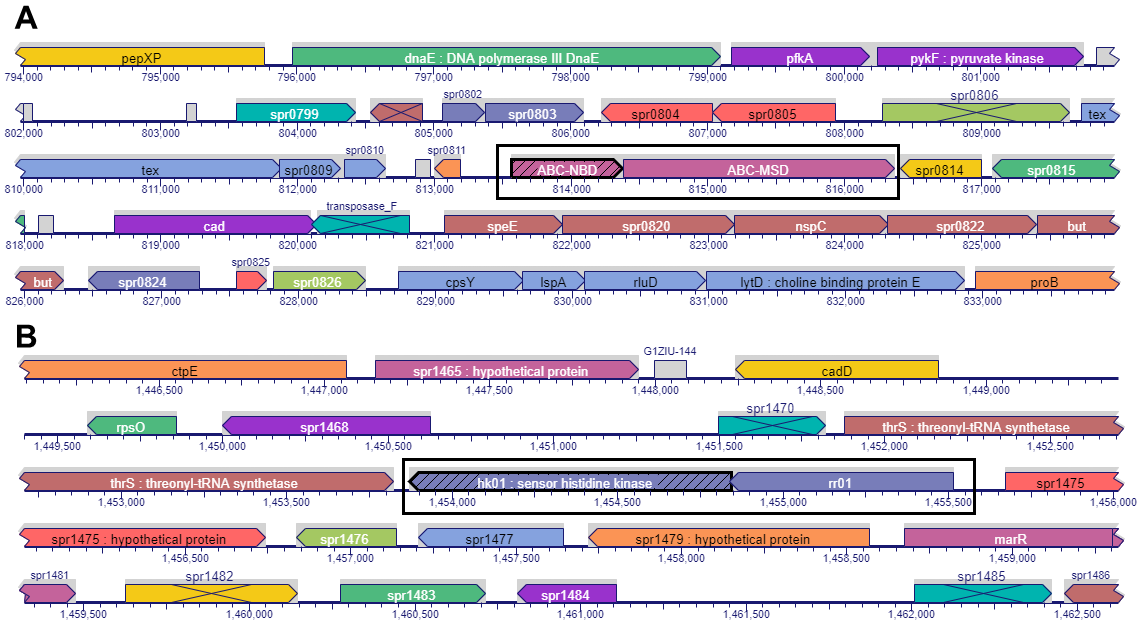

Supplement: S2 Fig — The genes of interest are highlighted in black boxes. This figure was prepared from data obtained in biocyc.org. (TIF) [file ppat.1010458.s006.tif]

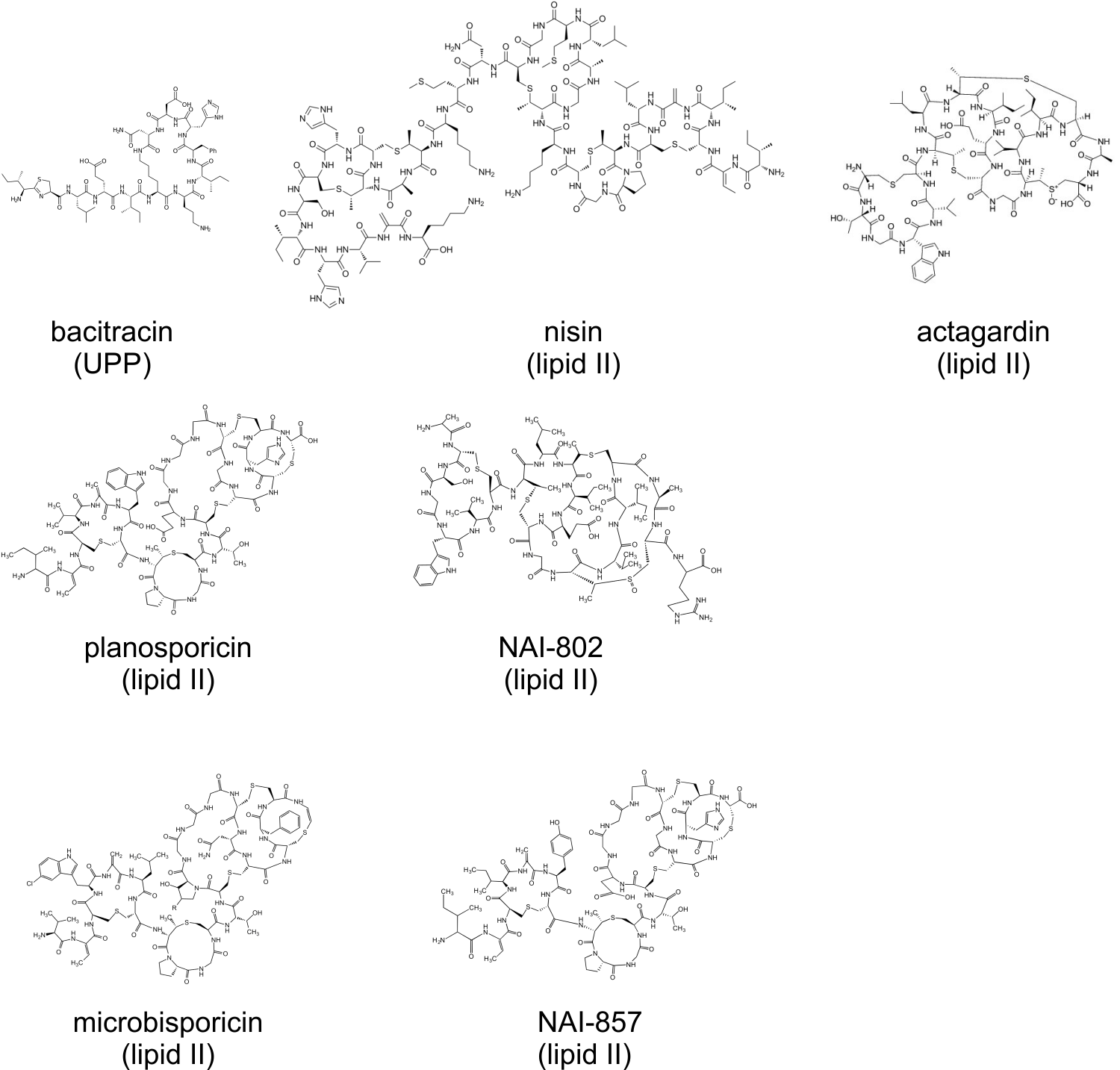

Supplement: S3 Fig — Only planosporicin, microbisporicin and NAI-857 are structurally related. (TIF) [file ppat.1010458.s007.tif]

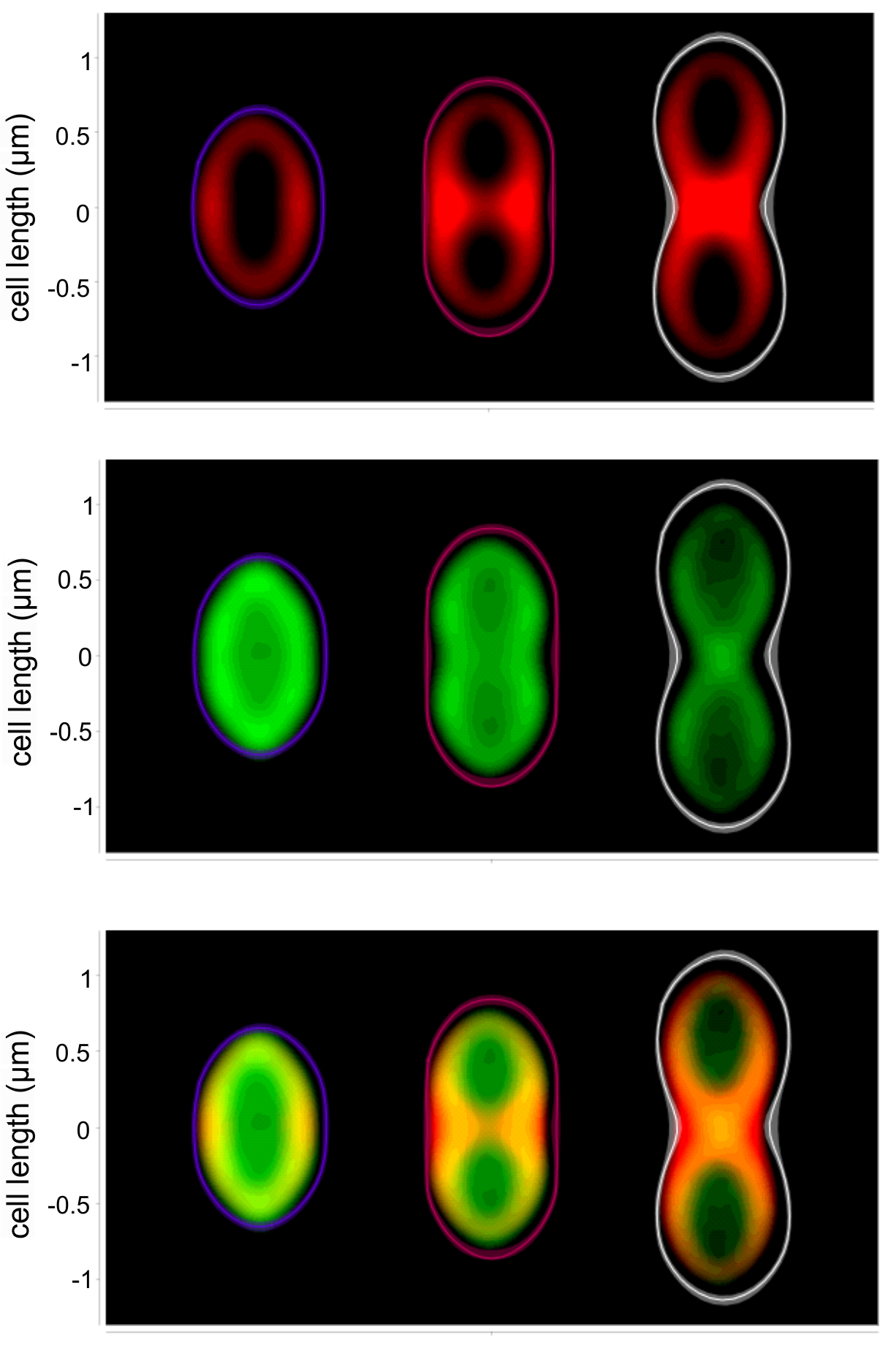

Supplement: S4 Fig — Cellular membranes were stained with 10 μg/mL of FM4-64 (top panel) and BceA-GFP fluorescence is shown in green (middle panel). The overlay is presented at the bottom panel, where the yellow color indicates the localization of BceA-GFP at the membrane. 2166 cells were analyzed in this representative experiment of a triplicate. (TIF) [file ppat.1010458.s008.tif]

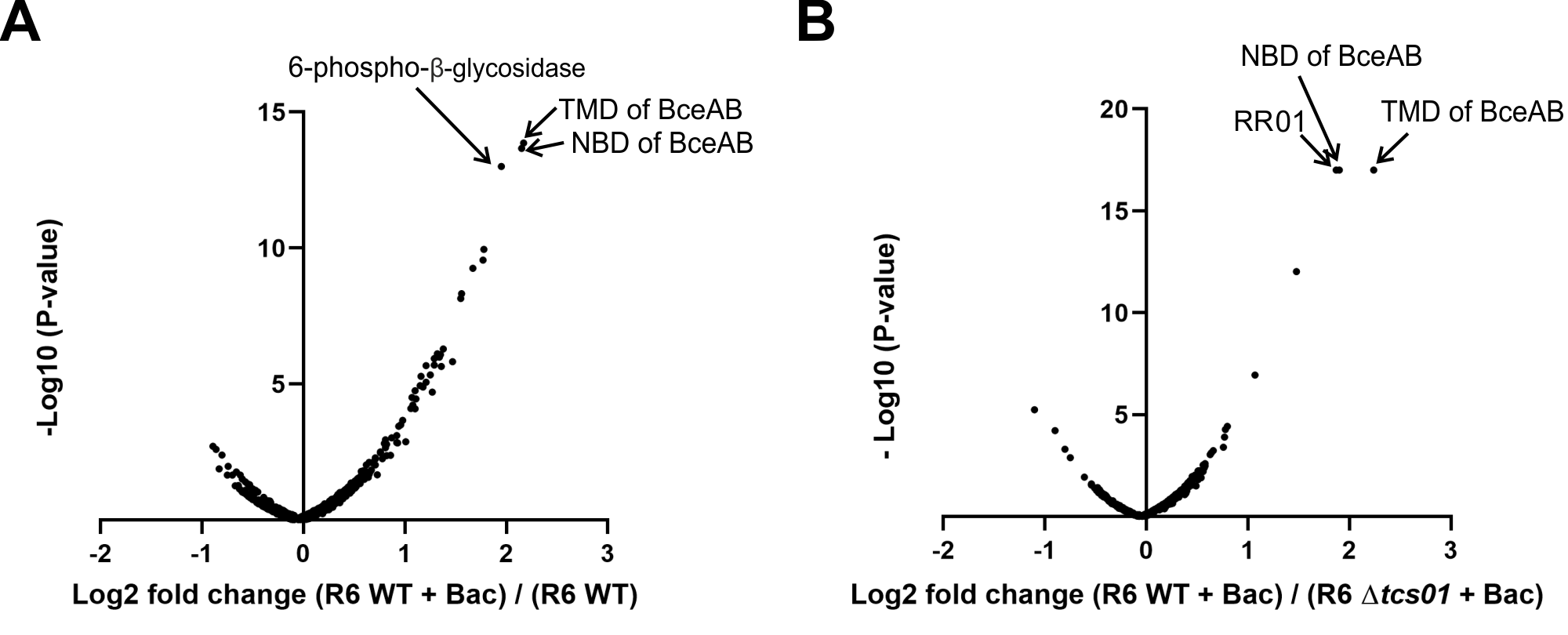

Supplement: S5 Fig — Data correspond to one biological replicate (experiment 1, see Material and Methods). A, volcano plot showing proteins differentially expressed in the wild-type strain upon bacitracin (Bac) treatment (1 μg/ml for 45 min). B, volcano plot showing proteins differentially expressed in wild-type strain as compared to the ΔTCS in the presence of bacitracin. In this experiment, the response regulator of RR01 was detected in the R6 WT + bacitracin sample and was artificially found overexpressed here due to the fact that it is deleted in the R6 Δtcs01 strain. Proteins are significantly overrepresented when Log2 (fold change) > 1 and–Log10 (P-value) > 1.3. Proteins are significantly underrepresented when Log2 (fold change) < -1 and–Log10 (P-value) > 1.3. (TIF) [file ppat.1010458.s009.tif]

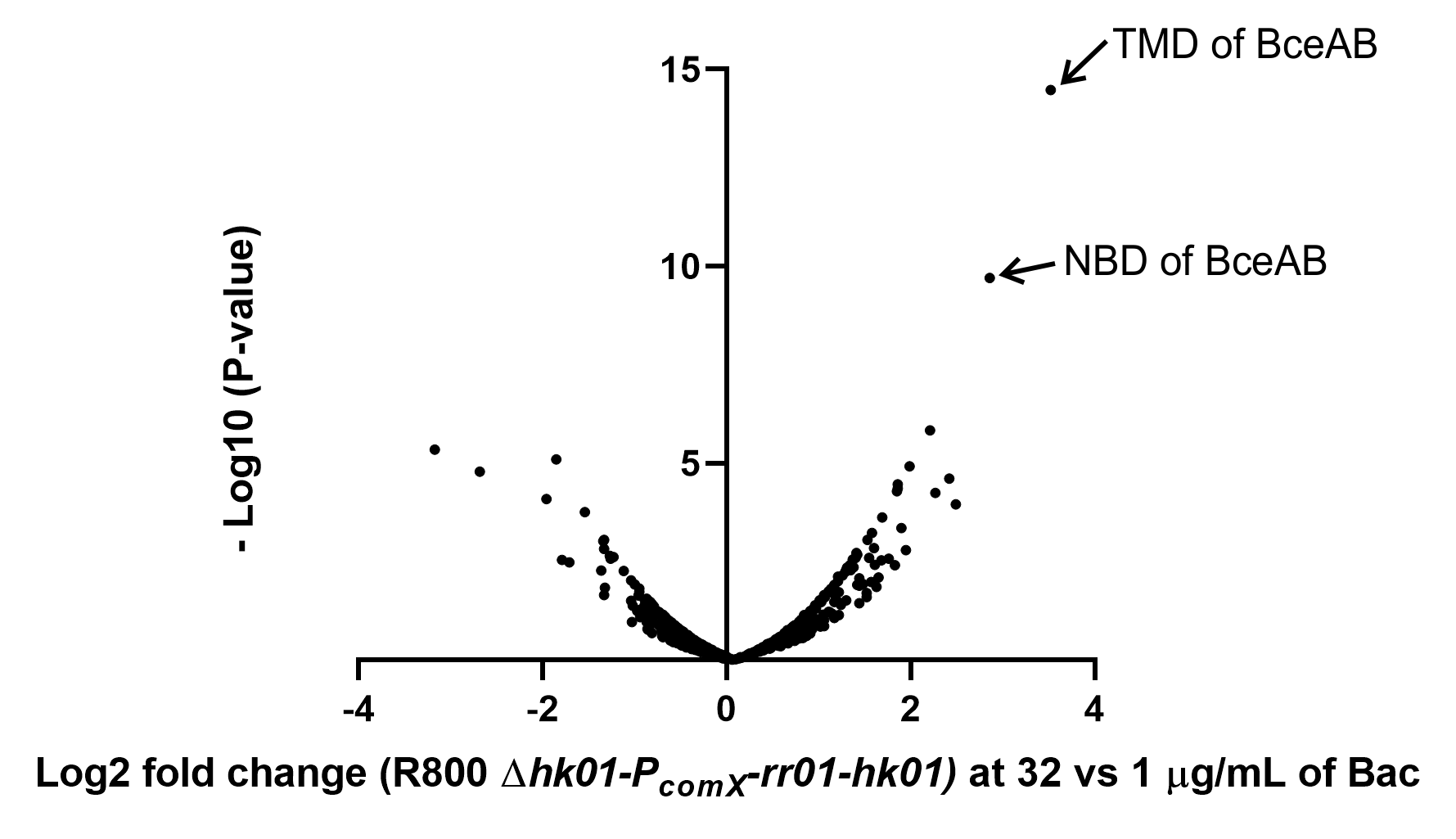

Supplement: S6 Fig — Volcano plot showing proteins differentially expressed in the R800-Δhk01-PcomX-hk01 strain upon differential bacitracin treatment (32 μg/ml vs 1 μg/ml for 45 min). Data correspond to the average of two biological replicates. Proteins are significantly overrepresented when Log2 (fold change) > 1 and–Log10 (P-value) > 1.3. Proteins are significantly underrepresented when Log2 (fold change) < -1 and–Log10 (P-value) > 1.3. (TIF) [file ppat.1010458.s010.tif]

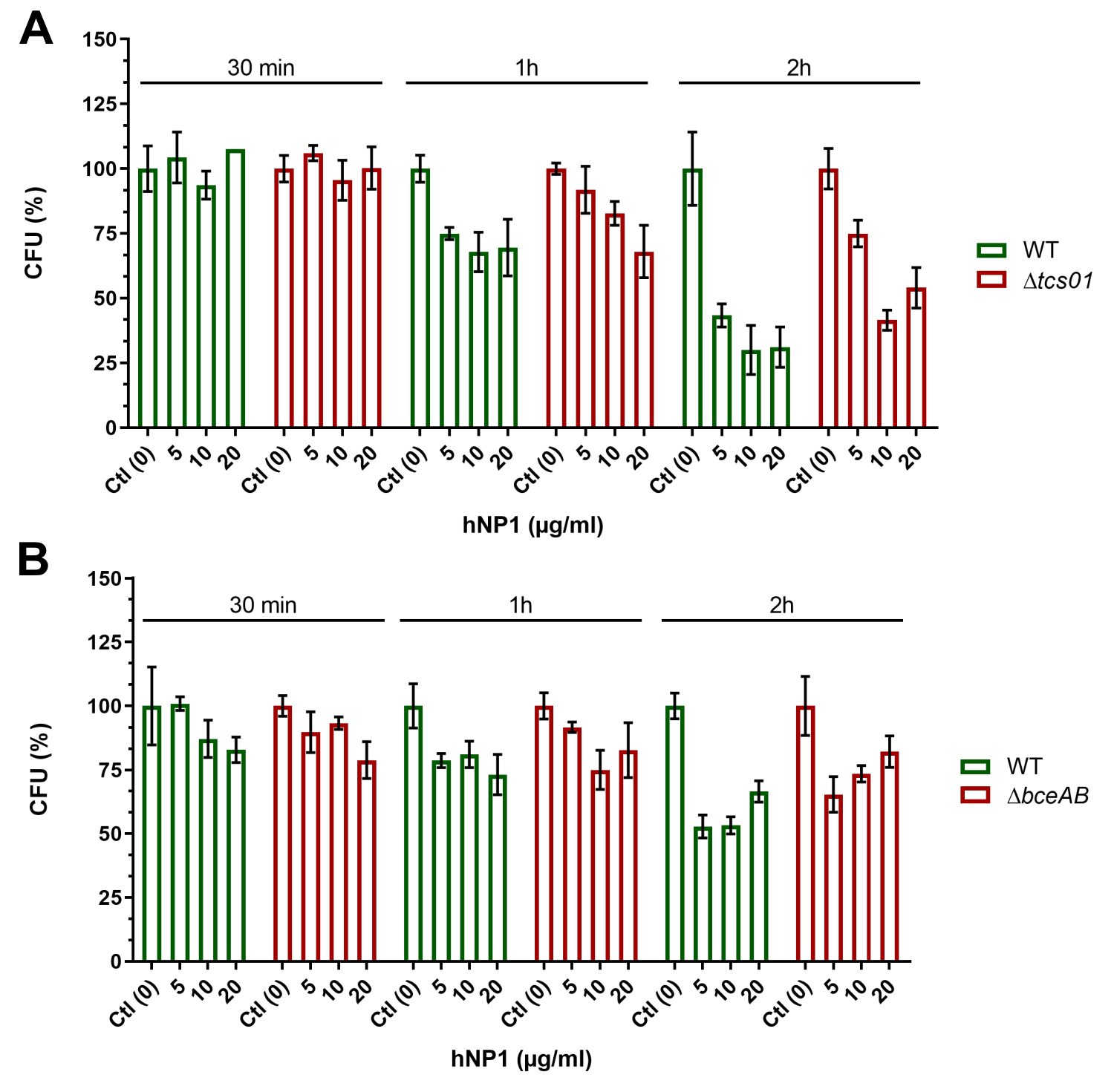

Supplement: S7 Fig — A, sensitivity of the WT and Δtcs01 strains to various concentrations of HNP1 over time. B, sensitivity of the WT and ΔbceAB strains to various concentrations of HNP1 over time. Data counts are the average of triplicates and were normalized according to the control cells (Ctl), at time zero in the absence of HNP1. (TIF) [file ppat.1010458.s011.tif]
